# Scaling up target regimens for tuberculosis preventive treatment in Brazil and South Africa: An analysis of costs and cost-effectiveness

**DOI:** 10.1371/journal.pmed.1004032

**Published:** 2022-06-13

**Authors:** Ntwali Placide Nsengiyumva, Jonathon R. Campbell, Olivia Oxlade, Juan F. Vesga, Christian Lienhardt, Anete Trajman, Dennis Falzon, Saskia Den Boon, Nimalan Arinaminpathy, Kevin Schwartzman

**Affiliations:** 1 McGill International Tuberculosis Centre, McGill University, Montreal, Quebec, Canada; 2 Respiratory Epidemiology and Clinical Research Unit, Montreal Chest Institute, Research Institute of the McGill University Health Centre, Montreal, Quebec, Canada; 3 Department of Epidemiology, Biostatistics, and Occupational Health, McGill University, Montreal, Quebec, Canada; 4 Department of Infectious Disease Epidemiology, Faculty of Medicine, Imperial College London, London, United Kingdom; 5 Institut de Recherche pour le Développement, Montpellier, France; 6 Programa de Pós-graduação em Clínica Médica, Federal University of Rio de Janeiro, Rio de Janeiro, Brazil; 7 Global TB Programme, World Health Organization, Geneva, Switzerland; 8 Respiratory Division, Department of Medicine, McGill University, Montreal, Quebec, Canada; Boston University School of Public Health, UNITED STATES

## Abstract

**Background:**

Shorter, safer, and cheaper tuberculosis (TB) preventive treatment (TPT) regimens will enhance uptake and effectiveness. WHO developed target product profiles describing minimum requirements and optimal targets for key attributes of novel TPT regimens. We performed a cost-effectiveness analysis addressing the scale-up of regimens meeting these criteria in Brazil, a setting with relatively low transmission and low HIV and rifampicin-resistant TB (RR-TB) prevalence, and South Africa, a setting with higher transmission and higher HIV and RR-TB prevalence.

**Methods and findings:**

We used outputs from a model simulating scale-up of TPT regimens meeting minimal and optimal criteria. We assumed that drug costs for minimal and optimal regimens were identical to 6 months of daily isoniazid (6H). The minimal regimen lasted 3 months, with 70% completion and 80% efficacy; the optimal regimen lasted 1 month, with 90% completion and 100% efficacy. Target groups were people living with HIV (PLHIV) on antiretroviral treatment and household contacts (HHCs) of identified TB patients. The status quo was 6H at 2019 coverage levels for PLHIV and HHCs. We projected TB cases and deaths, TB-associated disability-adjusted life years (DALYs), and costs (in 2020 US dollars) associated with TB from a TB services perspective from 2020 to 2035, with 3% annual discounting. We estimated the expected costs and outcomes of scaling up 6H, the minimal TPT regimen, or the optimal TPT regimen to reach all eligible PLHIV and HHCs by 2023, compared to the status quo. Maintaining current 6H coverage in Brazil (0% of HHCs and 30% of PLHIV treated) would be associated with 1.1 (95% uncertainty range [UR] 1.1–1.2) million TB cases, 123,000 (115,000–132,000) deaths, and 2.5 (2.1–3.1) million DALYs and would cost $1.1 ($1.0–$1.3) billion during 2020–2035. Expanding the 6H, minimal, or optimal regimen to 100% coverage among eligible groups would reduce DALYs by 0.5% (95% UR 1.2% reduction, 0.4% increase), 2.5% (1.8%–3.0%), and 9.0% (6.5%–11.0%), respectively, with additional costs of $107 ($95–$117) million and $51 ($41–$60) million and savings of $36 ($14–$58) million, respectively. Compared to the status quo, costs per DALY averted were $7,608 and $808 for scaling up the 6H and minimal regimens, respectively, while the optimal regimen was dominant (cost savings, reduced DALYs). In South Africa, maintaining current 6H coverage (0% of HHCs and 69% of PLHIV treated) would be associated with 3.6 (95% UR 3.0–4.3) million TB cases, 843,000 (598,000–1,201,000) deaths, and 36.7 (19.5–58.0) million DALYs and would cost $2.5 ($1.8–$3.6) billion. Expanding coverage with the 6H, minimal, or optimal regimen would reduce DALYs by 6.9% (95% UR 4.3%–95%), 15.5% (11.8%–18.9%), and 38.0% (32.7%–43.0%), respectively, with additional costs of $79 (−$7, $151) million and $40 (−$52, $140) million and savings of $608 ($443–$832) million, respectively. Compared to the status quo, estimated costs per DALY averted were $31 and $7 for scaling up the 6H and minimal regimens, while the optimal regimen was dominant. Study limitations included the focus on 2 countries, and no explicit consideration of costs incurred before the decision to prescribe TPT.

**Conclusions:**

Our findings suggest that scale-up of TPT regimens meeting minimum or optimal requirements would likely have important impacts on TB-associated outcomes and would likely be cost-effective or cost saving.

## Abstract 2

Placide Nsengiyumva and colleagues analyze costs and cost-effectiveness of scaling up target regimens for Tuberculosis Preventive Treatment among persons living with HIV and household contacts of TB patients in Brazil and South Africa.

## Author summary

**Why was this study done?:**

- As a key step toward tuberculosis (TB) elimination, the World Health Organization (WHO) has emphasized the expansion of tuberculosis preventive treatment (TPT) for persons with TB infection worldwide, beginning with those living with HIV and household contacts of persons with TB disease.
- The WHO has developed target product profiles describing minimum required characteristics and optimal (ideal but feasible) features of new regimens to treat TB infection.
- We sought to predict the net costs and cost-effectiveness of target regimens with minimal and optimal characteristics, in Brazil and South Africa: 2 settings with low versus high levels of TB transmission, HIV-TB coinfection, and rifampin-resistant TB.

**What did the researchers do and find?:**

- We used a calibrated, dynamic transmission model to predict TB-related morbidity, mortality, and costs from 2020 to 2035 associated with (1) continuation of the current TPT coverage (status quo) with a regimen of 6 months of daily isoniazid (6H) for persons who are living with HIV and receiving antiretroviral treatment or who are household contacts of identified TB patients, (2) expansion of the 6H regimen to cover all persons in these groups, and (3) similar expansion of regimens with either minimal or optimal features.
- In Brazil, the expansion of TPT regimens to 100% coverage among eligible groups would reduce TB-associated disability-adjusted life years (DALYs) modestly, by up to 9% for the optimal regimen. In South Africa, with higher TB transmission and higher HIV prevalence, expansion of these regimens would reduce TB-associated DALYs more substantially—by up to 38%.
- In both countries, TPT expansion with any of these regimens would likely be cost-effective based on established willingness-to-pay thresholds, while use of the optimal regimen would likely be cost saving.

**What do these findings mean?:**

- Expansion of TPT to better cover high-risk groups would likely be cost-effective. This would hold true even if drug costs were higher for novel regimens.
- Further development and implementation of novel TPT regimens should be pursued. Their impact on TB epidemiology and associated cost savings would be greatest in settings with high transmission and/or high HIV-TB coinfection prevalence.

## Introduction

One in 4 people worldwide is estimated to have evidence of tuberculosis (TB) infection [1]. People living with HIV (PLHIV) and household contacts (HHCs) of TB patients are at particularly high risk of progressing to TB disease [2–5]. The World Health Organization (WHO) End TB Strategy highlights the importance of expanded treatment of TB infection as a key step toward TB elimination, beginning with these priority groups. Despite important progress toward shorter and better tolerated TB preventive treatment (TPT) regimens, further improvements are needed to promote TPT uptake and implementation worldwide.

To support development of new TPT regimens, the WHO has described target product profiles (TPPs) listing minimum requirements and optimal (ideal but feasible) characteristics that would justify trials and adoption of novel regimens [6,7]. To inform the TPPs, epidemiological modeling for 4 high-burden countries was conducted to identify attributes that most influence the population impact of TPT. Regimens that are highly efficacious and easy to complete are projected to have major impact on the epidemic, particularly in high-transmission settings [8]. Building on this work, we conducted an economic evaluation of future TPT regimens that meet the minimum and optimal profile targets for priority attributes as proposed by the WHO. We considered the scale-up of “minimal” and “optimal” TPT regimens to meet WHO coverage targets in Brazil and South Africa, 2 of the 4 countries considered in the epidemiological modeling [8].

## Methods

This study followed the Consolidated Health Economic Evaluation Reporting Standards 2022 (CHEERS 2022) Statement [9] (see S1 CHEERS Checklist). All data underlying the analysis are available in S1 and S2 Datasets.

### Model overview

#### Epidemiological inputs

The model to derive epidemiological outcomes (i.e., TB incidence and TB mortality) is described in S1 Modeling Methods and detailed elsewhere [8]. Briefly, it is a deterministic, compartmental model calibrated to current WHO estimates for TB incidence, TB mortality, and TB-HIV coinfection. For the present economic evaluation, 2 countries were selected to represent highly distinct epidemiological settings: Brazil is a setting with relatively low TB transmission and low HIV and rifampicin-resistant TB (RR-TB) prevalence, while South Africa is a setting with high TB transmission and high HIV prevalence, with a substantial proportion of RR-TB [10].

The epidemiological model projected the impact of TPT on TB epidemiology among 2 subgroups in these countries: PLHIV initiating ART and all HHCs of notified TB patients. It considered a “status quo” scenario where current coverage with the TPT regimen of 6 months of daily isoniazid (6H) is maintained continuously from 2020 to 2035, with current coverage being 0% of HHCs in Brazil and South Africa receiving TPT, and 30% of PLHIV in Brazil and 69% of PLHIV in South Africa receiving TPT [10,11]. This time horizon was selected because the WHO End TB Strategy aims to end the global TB epidemic by 2035 [12]. The model compared this status quo scenario to expanding TPT using 6H or 1 of 2 novel regimens from 2020 to 2035. In these scale-up scenarios, it was assumed that TPT use met WHO targets: increasing linearly to 100% coverage for both PLHIV and HHCs over a 3-year period, with this level maintained thereafter until 2035 (see S1 Modeling Methods for more details).

For the 2 novel regimens, 5 attributes were considered as potential determinants of their impact during the TPP development process. These attributes were (1) duration of treatment; (2) efficacy against drug-susceptible TB (DS-TB) infection, defined as the reduction in incidence that would be observed under trial conditions at 2-year post-regimen follow-up; (3) barrier to rifampicin resistance, defined as the proportion of treated individuals with DS-TB infection who do not acquire RR-TB infection; (4) forgiveness for regimen non-completion, defined as the proportion of those who do not complete treatment (but take at least 50% of doses) who still receive full benefit; and (5) ease of adherence, defined as the proportion of patients who complete treatment under programmatic conditions. For each attribute, values were identified through an expert technical consultation convened by the WHO [7] and agreed upon by all 37 attendees to represent “minimal” and “optimal” novel regimens, identifying respectively the minimum acceptable performance of future regimens and feasible upper bounds (see Table 1). It was assumed that for the minimal and optimal regimens, efficacy against RR-TB infections was 50% lower than against DS-TB infections. For further details, see [6,8].

**Table 1:** Regimen attributes and input costs (in 2020 US dollars).

Parameter	6H	Minimal	Optimal
**Regimen attributes**			
Regimen duration (months)	6	3	1
Efficacy	70%	70%	100%
Rifampicin-resistance barrier	100%	95%	100%
Regimen forgiveness*	25%	50%	80%
Treatment completion	70%	80%	90%
**Cost inputs (derived by micro-costing)** ^**†**^			
Costs of medication per complete course of TPT regimen^a^	$3.70	$3.70	$3.70
Cost of visits, monitoring, and adverse events during TPT based on regimen characteristics^b^			
Brazil	$39.49	$24.85	$15.79
South Africa	$31.71	$19.62	$10.57

6H, 6 months of daily isoniazid; TPT, tuberculosis preventive treatment.

*Regimen forgiveness, defined as the proportion who receive full benefit among persons who complete between 50% and 100% of treatment.

^†^For references supporting the costs, please see Tables A–E in S1 File.

^a^Drug costs for the 2 novel regimens assumed to be equal to those of 6H monotherapy, as per WHO guidelines [13].

^b^For the minimal regimen, the rate of adverse events is assumed to be equivalent to that of 6H monotherapy. With the optimal regimen, it is assumed that there are no adverse events that require additional monitoring, hospitalization, or intervention.

The transmission model projected health outcomes over a 16-year time horizon (2020 to 2035) using 1,000 simulations. The primary outcomes were TB cases, TB-related deaths, and TB-associated disability-adjusted life years (DALYs); secondary outcomes included cumulative incidence of DS-TB and RR-TB. Outcomes were calculated by risk group (PLHIV and HHCs) and summed.

#### Cost inputs and outputs

We used a micro-costing approach to estimate costs for TPT and treatment of TB disease in Brazil and South Africa by itemizing use of health system personnel and resources for care of a typical patient (see Table 1). A detailed breakdown of component costs with supporting references is provided in Tables A–E in S1 File. All costs are expressed in 2020 US dollars using published inflation, purchasing power parity, and currency conversion indices [14,15].

In brief, TPT health system costs included an initial medical visit, treatment medication (assumed in the primary analysis to be equivalent to the cost of 6H in accordance with WHO guidelines [16]), and follow-up medical visits, the number of which was proportional to treatment duration. We assumed all TPT was self-administered. We calculated a weighted cost of TPT per patient based on the proportion completing versus not completing treatment, assuming those who did not complete treatment received 50% of doses on average. Costs of diagnosing TB infection or ruling out TB disease prior to TPT initiation were not considered, nor were any upstream training or infrastructure costs. For TB disease, costs included personnel time, diagnostic and monitoring tests, and TB medications. Health system costs related to TB disease were tabulated separately for patients with DS-TB and RR-TB disease. In our primary analysis, we assumed that 65% of TB disease treatment is administered via directly observed therapy (DOT) in Brazil, and 20% in South Africa, with the remainder being self-administered therapy (SAT)—i.e., combined SAT-DOT [17,18]. Costs for TB-related inpatient stays and outpatient visits and for monitoring treatment were obtained from national TB program data and the published literature (Tables A–E in S1 File). For both TB disease and TB infection, costs of treatment-related adverse events were prorated to their expected frequency. Costs of treating DS-TB disease and RR-TB disease were assumed to be independent of the TPT regimen used, with per person estimated DS-TB treatment costs of $999 and $562 in Brazil and South Africa, respectively, and RR-TB treatment costs of $12,457 and $10,199, respectively.

### Cost-effectiveness analysis

The cost-effectiveness analysis considered TPT- and TB-related costs from a TB services perspective. We fit all cost parameters to gamma distributions. We sampled probabilistically from each distribution 1,000 times to generate 1,000 cost estimates for TPT and treatment of DS-TB and RR-TB. Using the epidemiological outputs from the 1,000 runs of the transmission model, we applied these estimates to project country-level costs for TPT and care of TB disease from 2020 to 2035 in Brazil and South Africa. Cost and health outcomes presented in the primary analysis are discounted at 3% annually [19–21]. Costs were estimated for each country overall and stratified by risk group (PLHIV and HHCs). We estimated mean costs and outcomes for each strategy based on 1,000 model runs, with the 95% uncertainty range (UR) reflecting the 2.5th and 97.5th percentile estimates.

In our primary analysis for Brazil and South Africa, we performed 4 comparisons: (1) expanding 6H to meet WHO coverage targets versus status quo coverage with 6H; (2) expanding to WHO coverage targets using the minimal regimen versus status quo coverage with 6H; (3) expanding to WHO coverage targets with the minimal regimen versus expanded 6H; and (4) expanding to WHO coverage targets with the optimal regimen versus expanding with the minimal regimen. In each case, we estimated mean incremental costs or savings, and their 95% URs based on the 2.5th and 97.5th percentile estimates for those differences.

Where appropriate, we estimated (1) the incremental cost per DALY averted, (2) the incremental cost per TB case averted, and (3) the incremental cost per TB death averted. For the incremental cost per DALY averted, we considered a willingness-to-pay (WTP) threshold derived from published estimates of the health opportunity costs of health expenditures as they relate to DALYs averted in the 2 countries. For Brazil this value was $8,786 (in 2020 US dollars) per DALY averted, and for South Africa this value was $3,520 per DALY averted [22]. We plotted the estimated total cost and health outputs from all model simulations on cost-effectiveness planes.

#### Scenario and sensitivity analyses

We conducted several scenario and sensitivity analyses to assess the robustness of our results: (1) We considered an annual discount rate of 0% for cost and health outcomes, as well as 4%, which may be more reflective of economic growth in upper-middle-income countries [23]. (2) In a threshold sensitivity analysis, we varied the TPT medication cost to identify the break-even threshold for the minimal and optimal regimens as compared to 6H (i.e., the threshold drug cost at which the total predicted cost of TPT expansion using the novel regimen equals that of 6H expansion), and similarly for the optimal regimen compared to the minimal regimen. (3) We modeled a minimal regimen with varying barriers to the selection of rifampicin-resistant mutants, keeping other characteristics of the minimal regimen unchanged—we varied the barrier in 1% increments from 95% to 100% (i.e., a rifampicin-sparing regimen). (4) We considered alternate values for the efficacy of the minimal regimen against infection with RR-TB strains, changing this parameter from 50% in the base case to 25% and 75%. (5) We considered universal DOT (i.e., all patients receive DOT) as the standard for TB disease treatment. (6) We considered a longer time frame to reach full implementation of TPT among the target populations, extended from 3 years in the base case to 10 years and 16 years (the full duration of the simulation).

## Results

### Brazil

During 2020–2035, maintaining status quo 6H coverage (total of 669,000 persons treated) would result in a predicted 1.1 million TB cases, with 123,000 associated deaths and 2.5 million associated DALYs (see Table 2, which includes 95% URs). The total projected cost would be $1.1 billion. Expanding 6H to meet WHO targets would reduce TB mortality by 0.4% (95% UR 1.1% reduction, 0.6% increase) and DALYs by 0.5% (1.2% reduction, 0.4% increase), at an additional cost of $106 ($95–$117) million. Compared to the status quo, use of the minimal TPT regimen to meet WHO targets would reduce TB mortality by 2.7% (95% UR 2.1%–3.2%) and DALYs by 2.5% (1.8%–3.0%) at an additional cost of $51 ($41–$60) million. Use of the optimal regimen to meet WHO targets would reduce TB mortality by 8.4% (95% UR 6.4%–10.0%) and DALYs by 9.0% (6.5%–11.0%), with savings of $36 (95% UR $14–$58) million compared to the status quo.

**Table 2:** Projected health impacts and costs (95% uncertainty ranges) for TPT and TB treatment: Brazil 2020–2035.

Outcome	Status quo	Scale up 6H	Scale up minimal TPP regimen	Scale up optimal TPP regimen
**Health outcomes (thousands)**				
Number of people on TPT	669 (507–810)	3,721 (3,301–4,009)	3,712 (3,296–3,998)	3,694 (3,288–3,973)
TB cases (DS-TB and RR-TB)	1,128 (1,052–1,199)	1,124 (1,053–1,189)	1,095 (1,027–1,161)	1,039 (978–1,096)
TB deaths	123 (115–132)	123 (115–131)	120 (112–128)	113 (107–120)
TB DALYs	2,542 (2,101–3,118)	2,528 (2,087–3,101)	2,479 (2,044–3,044)	2,313 (1,913–2,859)
**Cost estimates (millions of US dollars)**				
Cost of TPT	$25 ($19–$30)	$134 ($118–$148)	$90 ($79–$99)	$62 ($55–$69)
Cost for TB disease management	$1,116 ($986–$1,265)	$1,112 ($990–$1,256)	$1,102 ($979–$1,249)	$1,042 ($933–$1,177)
Total cost (TPT and TB disease)	$1,140 ($1,009–$1,291)	$1,247 ($1,119–$1,400)	$1,191 ($1,064–$1,346)	$1,104 ($991–$1,243)

6H, 6 months of daily isoniazid; DALY, disability-adjusted life year; DS-TB, drug-susceptible tuberculosis; RR-TB, rifampicin-resistant tuberculosis; TB, tuberculosis; TPP, target product profile; TPT, tuberculosis preventive treatment.

Compared to expanding 6H, expanding use of the minimal regimen would be associated with 29,000 (95% UR 24,000–35,000) fewer cases, 2,800 (2,300–3,400) fewer deaths, and 49,000 (36,000–66,000) more DALYs averted. The minimal regimen would result in $55 ($45–$65) million in savings compared to expanding 6H. The optimal regimen would avert a further 56,000 (95% UR 41,000–71,000) TB cases, 9,800 (7,700–11,800) deaths, and 215,000 (152,000–282,000) DALYs, and generate an additional $87 ($69–$105) million in savings compared to the minimal regimen. Benefits and savings were greater among PLHIV than HHCs. More detailed breakdowns of health outcomes and costs, as well as outcomes stratified by PLHIV and HHCs, are provided in Tables F–I in S1 File.

**Fig 1:**
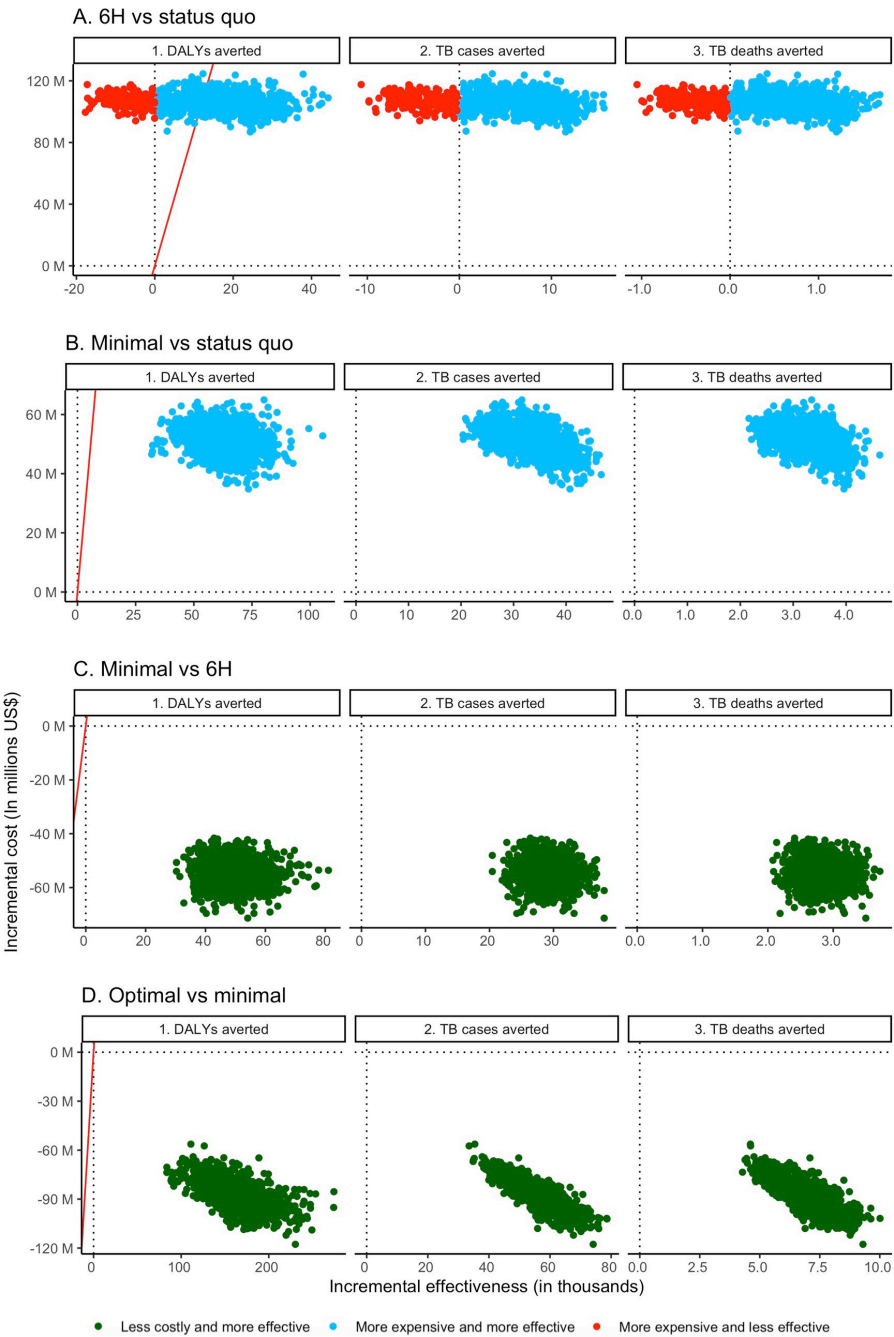
Brazil incremental cost-effectiveness planes. (A) Six months of daily isoniazid (6H) versus status quo. (B) Minimal regimen versus status quo. (C) Minimal regimen versus 6H. (D) Optimal versus minimal regimen. On the *x*-axis, negative values indicate poorer health outcomes. On the *y*-axis, negative values indicate cost savings. The red lines correspond to the willingness-to-pay threshold per disability-adjusted life year (DALY) averted ($8,786/DALY averted). TB, tuberculosis.

When 6H expansion was compared to status quo 6H coverage, we projected incremental costs of $22,437 per TB case averted, $204,243 per TB death averted, and $7,608 per DALY averted. In probabilistic analysis, expanding 6H was more expensive but averted more TB cases and more TB deaths in 79% and 80% of simulations, respectively; it averted more DALYs in 84% of simulations (Fig 1A). We predicted that expanding 6H would fall below the WTP threshold for DALYs averted in 60% of simulations (Fig 1A).

Compared to status quo 6H coverage, using the minimal TPT regimen to reach WHO targets cost an estimated $1,509 per TB case averted, $15,328 per TB death averted, and $808 per DALY averted. In probabilistic analysis, the minimal regimen was associated with higher cost and better health outcomes than status quo 6H coverage in all simulations (Fig 1B). It was consistently associated with incremental costs per DALY averted below the WTP threshold. When comparing the minimal regimen to expanded 6H coverage to reach WHO targets, the minimal regimen was dominant—it was associated with improved health outcomes and cost savings in all simulations (Fig 1C).

Compared to the minimal TPT regimen, the optimal TPT regimen was dominant—resulting in improved health outcomes and cost savings in all simulations (Fig 1D).

### South Africa

Overall projected TB-related morbidity, mortality, and costs in South Africa were much higher than in Brazil, as expected. Continued status quo 6H coverage during 2020–2035 (total of 4.1 million persons treated) would result in 3.7 million TB cases, 843,000 TB deaths, and 36.7 million DALYS (see Table 3, which includes 95% URs). The total projected cost would be $2.5 billion. Expanding 6H to meet WHO targets would reduce TB mortality by 6.5% (95% UR 4.0%–9.0%) and DALYs by 6.9% (4.3%–9.5%), at an additional cost of $79 (−$7, $151) million. Compared to the status quo, use of the minimal TPT regimen to meet WHO targets would reduce TB mortality by 14.8% (95% UR 11.1%–18.1%) and DALYs by 15.5% (11.8%–18.9%) at an additional cost of $41 (−$52, $140) million. Use of the optimal regimen to meet WHO targets would reduce TB mortality by 36.8% (95% UR 31.7%–41.8%) and DALYs by 38.0% (32.7%–43.0%), with savings of $608 ($443–$832) million compared to the status quo.

**Table 3:** Projected health impacts and costs (95%uncertainty ranges) for TPT and TB treatment: South Africa 2020–2035.

Outcome	Status quo	Scale up 6H	Scale up minimal TPP regimen	Scale up optimal TPP regimen
**Health outcomes (thousands)**				
Number of people on TPT	4,109 (3,252–4,852)	10,135 (8,521–11,417)	10,071 (8,516–11,338)	9,920 (8,454–11,049)
TB cases (DS-TB and RR-TB)	3,663 (3,002–4,393)	3,398 (2,800–4,023)	3,015 (2,477–3,600)	2,164 (1,826–2,517)
TB deaths	843 (548–1,201)	788 (518–1,122)	718 (470–1,034)	531 (359–752)
TB DALYs	36,682 (19,459–58,024)	34,159 (17,903–54,184)	31,067 (15,946–49,928)	22,736 (11,995–35,847)
**Cost estimates (millions of US dollars)**				
Cost of TPT	$145 ($113–$172)	$349 ($290–$401)	$248 ($207–$285)	$172 ($144–$196)
Cost for TB disease management	$2,391 ($1,656–$3,414)	$2,265 ($1,575–$3,233)	$2,328 ($1,588–$3,356)	$1,733 ($1,182–$2,614)
Total cost (TPT and TB disease)	$2,535 ($1,801–$3,560)	$2,614 ($1,922–$3,580)	$2,576 ($1,840–$3,624)	$1,927 ($1,353–$2,786)

6H, 6 months of daily isoniazid; DALY, disability-adjusted life year; DS-TB, drug-susceptible tuberculosis; RR-TB, rifampicin-resistant tuberculosis; TB, tuberculosis; TPP, target product profile; TPT, tuberculosis preventive treatment.

Compared to expanded 6H, the minimal regimen was associated with 384,000 (95% UR 282,000–494,000) fewer TB cases, 69,000 (46,000–94,000) fewer deaths, and 3.1 (1.8–4.5) million DALYs averted. The minimal regimen would result in $38 million in savings (95% UR $109 million in savings, $70 million in additional cost) compared to expanded 6H. The optimal regimen would avert a further 851,000 (95% UR 641,000, 1.1 million) TB cases, 187,000 (107,000–291,000) deaths, and 8.3 (4.0–14.0) million DALYs, and generate an additional $649 ($457–$903) million in savings compared to the minimal regimen. As in Brazil, benefits and savings were greater among PLHIV than among HHCs. More detailed breakdowns of health outcomes and costs, as well as outcomes stratified by PLHIV and HHCs, are provided in Tables F–I in S1 File.

**Fig 2:**
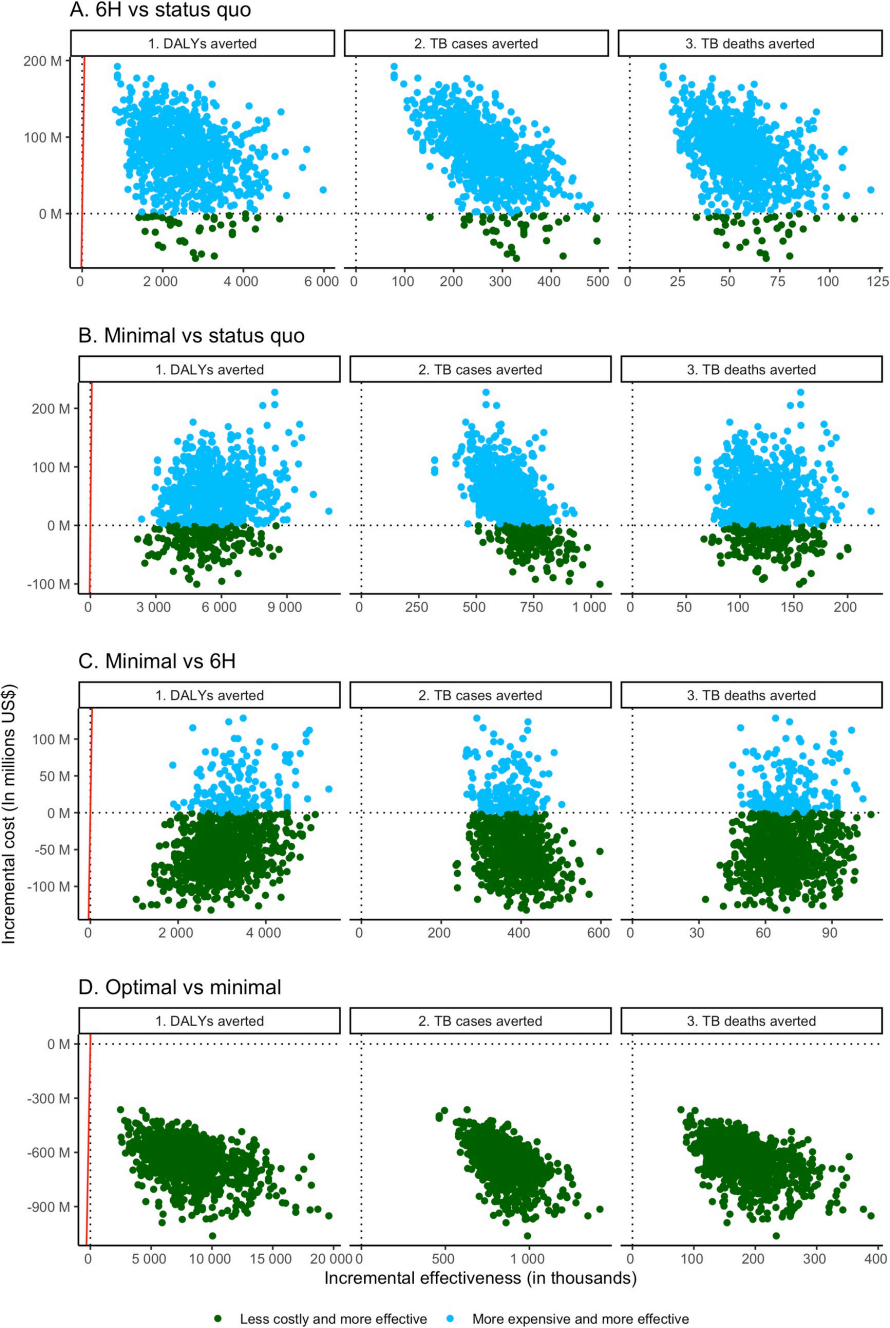
South Africa incremental cost-effectiveness planes. (A) Six months of daily isoniazid (6H) versus status quo. (B) Minimal regimen versus status quo. (C) Minimal regimen versus 6H. (D) Optimal versus minimal regimen. On the *y*-axis, negative values indicate cost savings. The red lines correspond to the willingness-to-pay threshold per disability-adjusted life year (DALY) averted ($3,520/DALY averted). TB, tuberculosis.

When expanding 6H to WHO targets was compared to status quo 6H coverage, we projected incremental costs of $297 per TB case averted, $1,426 per TB death averted, and $31 per DALY averted. In probabilistic analysis, expanding 6H was associated with improved health outcomes in all simulations, and additional cost in 96% of simulations (Fig 2A). In all simulations, expanding 6H fell below the WTP threshold for DALYs averted relative to the status quo.

Compared to status quo 6H coverage, using the minimal TPT regimen to reach WHO targets would cost an estimated $62 per TB case averted, $326 per TB death averted, and $7 per DALY averted. In probabilistic analysis, the minimal regimen was associated with higher cost in 79% of simulations and better health outcomes in all simulations compared to status quo 6H coverage (Fig 2B). It was consistently associated with incremental costs per DALY averted that fell well below the WTP threshold. Compared to expanding 6H coverage, use of the minimal TPT regimen to meet WHO targets was dominant in 81% of simulations, associated with improved health outcomes and lower costs (Fig 2C). In all other simulations, the minimal TPT regimen was associated with improved health and fell below the WTP threshold for incremental cost per DALY averted relative to expanding 6H coverage.

The optimal TPT regimen was dominant compared to the minimal TPT regimen; it was associated with marked improvement in health outcomes, as well as cost savings, in all simulations (Fig 2D).

### Scenario and sensitivity analyses

Analyses without discounting and using a 4% discount rate yielded similar findings to the primary analysis with respect to WTP thresholds per DALY averted (Tables J and K and Figs A–D in S1 File).

In break-even threshold analyses with varying medication costs for the minimal and optimal TPT regimens, use of the minimal regimen to meet WHO targets in Brazil became more expensive than expanding 6H when medication costs were at least $26, while use of the optimal regimen became more costly when medication costs were at least $53; the optimal regimen became more costly than the minimal regimen when medication costs were at least $35. In South Africa, the corresponding thresholds were $27 for the minimal regimen and $142 for the optimal regimen when compared to expanded 6H, and $122 for the optimal regimen compared to the minimal regimen. See Figs E and F in S1 File for further detail.

Varying the RR-TB barrier from 95% to 100% had little effect on expected RR-TB cases and total costs (Figs G and H in S1 File). Similarly, the assumed efficacy of TPT regimens against infection with rifampin-resistant strains had minimal impact on expected RR-TB cases and total costs, when changed from 50% in the base case to 25% or 75% (Figs I and J in S1 File).

In a scenario analysis that assumed 100% DOT use for TB disease treatment, we projected similar findings, except that the minimal TPT regimen became cost saving compared to status quo 6H coverage in South Africa. This reflected the higher cost of DOT and thus greater absolute savings per TB case averted, compared to combined SAT-DOT. Detailed cost estimates are shown in Tables L and M in S1 File.

Analyses of delayed scale-up of TPT regimens from 3 years to 10 or 16 years did not qualitatively impact the cost-effectiveness of any regimen implemented in South Africa (Tables N and O and Figs K–N in S1 File). In Brazil, however, expansion of 6H compared to the status quo was associated with costs per DALY averted above the WTP threshold, with estimates of $9,670 and $10,509 when scaled over 10 years and 16 years, respectively (Tables N and O and Figs O–R in S1 File). Other comparisons in Brazil were not qualitatively impacted by delayed scale-up.

## Discussion

In this analysis, we considered the potential costs and cost-effectiveness of novel regimens for TPT to inform WHO TPP development. Expanding TPT coverage with improved regimens is likely to be highly cost-effective or even cost saving, while providing important health gains. Though this was true for a setting with low transmission, HIV prevalence, and RR-TB incidence as well as for one with high transmission, HIV prevalence, and RR-TB incidence, health gains and cost savings were greater in the latter, exemplified by South Africa.

Shorter regimens entail fewer provider visits and likely have improved completion, as observed for all shorter course TPT regimens thus far [24,25]. Although the cost of novel regimens remains uncertain at this time, we considered a wide range of potential values. Even if the drugs themselves are more expensive, improved regimens will likely remain cost saving or cost-effective. In our primary analysis, we considered that treatment for TB disease involved a mixture of SAT and DOT. Even under different care delivery assumptions, novel regimens remained highly cost-effective or cost saving. A strength is our use of recent estimates for WTP thresholds per DALY averted specific to Brazil and South Africa. These are based on observed health opportunity costs and outcomes in those countries [22], and are generally lower than other WTP threshold metrics [26,27]. In addition, the cost per TB case averted by the minimal regimen in Brazil was only slightly higher than the average cost of treating a case of TB ($1,507 versus $1,285), while in South Africa the cost per TB case averted was much lower than the TB treatment cost ($62 versus $919); in both countries the optimal regimen was associated with a cost per TB case averted that was much lower than the cost of treating TB disease. In this context, the robustness of our findings is especially reassuring.

We did not explicitly consider upfront research and development (R&D) costs for newer regimens [28,29]. However, these will vary depending on whether they involve new molecules, or new uses (or combinations) of existing agents. In the case of proprietary medications, R&D costs will likely be integrated into purchase costs for TB programs. It is also possible that some R&D and purchase costs will be borne by international donors. The present analysis suggests substantial economic and health benefits would result from investments by both national programs and international donors in the development of shorter, more effective TPT regimens.

Our analysis focused on future regimens, but also considered expansion of the current standard of care regimen, 6H. The “minimal” regimen profile most closely resembles the currently available 3-month isoniazid-rifapentine regimen (estimated cost of $29 and $66 in Brazil and South Africa, respectively [30]) with respect to duration, completion, and probable efficacy. In 2 countries representing distinct epidemiological settings, use of the minimal regimen for PLHIV and HHCs was highly cost-effective, or even cost saving, compared with expanding 6H. This reflects improved completion, as well as better forgiveness of missed doses. The optimal regimen—with even shorter duration, better efficacy and completion, and rare need for monitoring—will clearly provide further cost savings and health gains in diverse epidemiological settings, primarily related to improved efficacy.

As with any analysis based on modeling, results may be limited by uncertainty related to relevant epidemiological and cost parameters. We considered a wide range of regimen parameters, using probabilistic analyses as well as several scenario analyses. These also accounted for uncertainty in epidemiological predictions [8]. The analysis considered administrative and overhead costs only in direct proportion to treatment initiations, and hence did not specifically address training, administrative, or oversight costs related to program expansion. However, as the interventions were cost saving or highly cost-effective, there is likely a great deal of investment that could be made to expand programs before interventions would become cost-prohibitive.

We did not consider the costs of identifying PLHIV and HHCs with TB infection. These would not vary between TPT regimens; as with training and administration, these costs would be common to all scale-up scenarios. Nonetheless, expanding TPT to all HHCs, compared to expanding TPT to PLHIV already linked to care through ART programs, would be more challenging from a logistic and cost perspective. While costs of identifying PLHIV linked to ART programs may be small, costs associated with identifying HHCs are likely to be much greater because this process requires HHC investigations. Reassuringly, a previous analysis of the 3-month isoniazid and rifapentine regimen (similar to the minimal regimen) suggested that consideration of the costs associated with the identification of HHCs would be unlikely to make this intervention cost-prohibitive [30].

Our analysis also reflected the optimistic assumption that scale-up could be accomplished over a 3-year period—an ambitious goal that will be more challenging in the face of the COVID-19 pandemic, which was not considered in the present analysis. We found that longer durations of scale-up did not qualitatively impact our findings for scale-up of the minimal and optimal regimens in either setting, nor for 6H expansion in South Africa. However, we found that the cost-effectiveness of 6H scale-up in Brazil was impacted by longer time to full implementation, with costs per DALY averted slightly above the WTP threshold for both 10- and 16-year time frames.

As more solid clinical data emerge for new regimens, it will be relevant to incorporate these data into this type of analysis as TPT is expanded, first to the established priority groups and then to other people. Future analyses can also address additional expansion of TPT to different epidemiological settings and populations. Clearly, progress toward global TB elimination will ultimately require use of TPT beyond PLHIV and HHCs. Optimal regimen characteristics will become more important as TPT use is expanded to groups at lower risk of TB disease, which will result in increasing numbers of persons needing treatment to prevent each additional TB case [31]. Conversely, improved biomarkers and models to predict progression to TB disease would allow better targeting of TPT, with attendant gains in cost-effectiveness [32]. In all scenarios, however, overall reductions in TB incidence will reduce transmission as well as the total budgetary impact of TPT in the longer term.

In conclusion, this analysis provides evidence for substantial epidemiological and economic benefits of expanding TPT using existing regimens and new regimens fulfilling WHO recommended attributes [6,33–37]. Beyond the additional health gains and savings from providing improved regimens to PLHIV and HHCs, it supports the need for ongoing investment in R&D for novel regimens and in TB-related diagnostic and treatment infrastructure. More importantly, it suggests that the use of novel TPT regimens meeting minimal and optimal profiles envisaged in the WHO TPPs can potentially reduce TB-related costs as well as TB-related morbidity and mortality, particularly in settings with high TB transmission and/or high HIV prevalence. While we await development and implementation of these new regimens, expanding coverage of existing regimens is likely to be beneficial for populations and cost-effective for health systems.

## Supporting information

S1 CHEERS Checklist Consolidated Health Economic Evaluation Reporting Standards 2022 checklist. (PDF)

S1 Dataset Undiscounted costs and outcomes from all analyses. (XLSX)

S2 Dataset Costs and outcomes from all analyses discounted at 3%. (XLSX)

S1 File Contains details of cost inputs (Tables A–E), health and cost outcomes (Tables F–I), and additional results (Tables J–O and Figs A–R). Table A. Health system cost—programmatic management of TPT—current standard of care. Table B. Regimens for TPT, DS-TB, and RR-TB—Brazil. Table C. Regimens for TPT, DS-TB, and RR-TB—South Africa. Table D. Health system cost—drug-susceptible TB disease diagnosis and treatment—current standard of care. Table E. Health system cost—RR-TB disease treatment—current standard of care. Table F. Epidemiological estimates (95% uncertainty ranges [URs]) (in thousands) at 3% annual discount rate, Brazil and South Africa, 2020–2035. Table G. Incremental effectiveness (in thousands) at 3% annual discount rate, Brazil and South Africa, 2020–2035. Table H. Cost estimates (95% URs) for TPT and for TB disease treatment by combined SAT-DOT at 3% annual discount rate, Brazil and South Africa, 2020–2035 (US dollars in millions). Table I. Incremental costs (95% URs) for TPT and for TB disease treatment by combined SAT-DOT at 3% annual discount rate, Brazil and South Africa, 2020–2035 (US dollars in millions). Table J. Cumulative effectiveness projections at a 4% annual discount rate (in thousands), Brazil and South Africa, 2020–2035. Table K. Cumulative undiscounted effectiveness projections (in thousands), Brazil and South Africa, 2020–2035. Fig A. Incremental undiscounted costs, Brazil. Fig B. Incremental costs discounted at a 4% annual rate, Brazil. Fig C. Incremental undiscounted costs, South Africa. Fig D. Incremental costs discounted at a 4% annual rate, South Africa. Fig E. Threshold analysis of TPT, Brazil—break-even point stratified by treatment group. Fig F. Threshold analysis of TPT, South Africa—break-even point stratified by treatment group. Fig G. Varying the rifampicin-resistance barrier, Brazil, 2020–2035. Fig H. Rifampicin-resistance barrier, South Africa, 2020–2035. Fig I. Varying the effect of TPT regimens on rifampin-resistant strains, Brazil. Fig J. The effect of TPT regimens on rifampin-resistant strains, South Africa. Table L. Cost estimates (95% URs) for TPT and for TB disease treatment by universal DOT at 3% annual discount rate, Brazil and South Africa, 2020–2035 (US dollars in millions). Table M. Incremental costs (95% URs) for TPT and for TB disease treatment by universal DOT at 3% annual discount rate, Brazil and South Africa, 2020–2035 (US dollars in millions). Fig K. Incremental costs, 10-year scale-up of TPT, South Africa. Fig L. Incremental costs, 16-year scale-up of TPT, South Africa. Fig M. Incremental cost-effectiveness planes, 10-year scale-up of TPT, South Africa. Fig N. Incremental cost-effectiveness planes, 16-year scale-up of TPT, South Africa. Table N. Cumulative effectiveness projections with a 10-year scale-up of TPT (in thousands), Brazil and South Africa, 2020–2035. Table O. Cumulative effectiveness projections with a 16-year time horizon scale-up of TPT (in thousands), Brazil and South Africa, 2020–2035. Fig O. Incremental costs, 10-year scale-up of TPT, Brazil. Fig P. Incremental costs, 16-year scale-up of TPT, Brazil. Fig Q. Incremental cost-effectiveness planes, 10-year scale-up of TPT, Brazil. Fig R. Incremental cost-effectiveness planes, 16-year scale-up of TPT, Brazil.
(DOCX)

S1 Modeling Methods Detailed description of mathematical model and associated parameters. (DOCX)

## Abbreviations

6H: 6 months of daily isoniazid
DALY: disability-adjusted life year
DOT: directly observed therapy
DS-TB: drug-susceptible tuberculosis
HHC: household contact
PLHIV: people living with HIV
R&D: research and development
RR-TB: rifampicin-resistant tuberculosis
SAT: self-administered therapy
TB: tuberculosis
TPP: target product profile
TPT: tuberculosis preventive treatment
UR: uncertainty range
WHO: World Health Organization
WTP: willingness-to-pay
