# Supplementary material for: Scaling up target regimens for tuberculosis preventive treatment in Brazil and South Africa: An analysis of costs and cost-effectiveness
Source: PLoS Med. 2022 Jun 13;19(6):e1004032. doi: 10.1371/journal.pmed.1004032 (PMC9239450; doi:10.1371/journal.pmed.1004032)
Supplement: S1 File — Contains details of cost inputs (Tables A–E), health and cost outcomes (Tables F–I), and additional results (Tables J–O and Figs A–R). Table A. Health system cost—programmatic management of TPT—current standard of care. Table B. Regimens for TPT, DS-TB, and RR-TB—Brazil. Table C. Regimens for TPT, DS-TB, and RR-TB—South Africa. Table D. Health system cost—drug-susceptible TB disease diagnosis and treatment—current standard of care. Table E. Health system cost—RR-TB disease treatment—current standard of care. Table F. Epidemiological estimates (95% uncertainty ranges [URs]) (in thousands) at 3% annual discount rate, Brazil and South Africa, 2020–2035. Table G. Incremental effectiveness (in thousands) at 3% annual discount rate, Brazil and South Africa, 2020–2035. Table H. Cost estimates (95% URs) for TPT and for TB disease treatment by combined SAT-DOT at 3% annual discount rate, Brazil and South Africa, 2020–2035 (US dollars in millions). Table I. Incremental costs (95% URs) for TPT and for TB disease treatment by combined SAT-DOT at 3% annual discount rate, Brazil and South Africa, 2020–2035 (US dollars in millions). Table J. Cumulative effectiveness projections at a 4% annual discount rate (in thousands), Brazil and South Africa, 2020–2035. Table K. Cumulative undiscounted effectiveness projections (in thousands), Brazil and South Africa, 2020–2035. Fig A. Incremental undiscounted costs, Brazil. Fig B. Incremental costs discounted at a 4% annual rate, Brazil. Fig C. Incremental undiscounted costs, South Africa. Fig D. Incremental costs discounted at a 4% annual rate, South Africa. Fig E. Threshold analysis of TPT, Brazil—break-even point stratified by treatment group. Fig F. Threshold analysis of TPT, South Africa—break-even point stratified by treatment group. Fig G. Varying the rifampicin-resistance barrier, Brazil, 2020–2035. Fig H. Rifampicin-resistance barrier, South Africa, 2020–2035. Fig I. Varying the effect of TPT regimens on rifampin-resistant strains, B [file pmed.1004032.s004.docx]

**Scaling-up Target Regimens for Tuberculosis Preventive Treatment in Brazil and South Africa: An Analysis of Costs and Cost-Effectiveness**

**Supplementary appendix**

[**Table A**. Health system cost – programmatic management of TPT - Current Standard of Care 4](#_Toc104819125)

[**Table B.** Regimens for TPT, DS-TB and RR-TB -Brazil 4](#_Toc104819126)

[**Table C.** Regimens for TPT, DS-TB and RR-TB – South Africa 5](#_Toc104819127)

[**Table D**. Health system cost – drug-susceptible TB disease diagnosis and treatment - Current Standard of Care 6](#_Toc104819128)

[**Table E**. Health system cost -- RR-TB disease treatment - Current standard of care 7](#_Toc104819129)

[**Table F.** Epidemiological estimates (95% uncertainty ranges, UR) (in thousands) at 3% annual discount rate, Brazil and South Africa, 2020–2035 8](#_Toc104819130)

[**Table G.** Incremental effectiveness (in thousands) at 3% annual discount rate, Brazil and South Africa, 2020-2035 10](#_Toc104819131)

[**Table H.** Cost estimates (95% UR) for TPT, and for TB disease treatment by combined SAT-DOT at 3% annual discount rate, Brazil and South Africa, 2020-2035 (US dollars in Millions) 12](#_Toc104819132)

[**Table I**. Incremental costs (95% UR) for TPT, and for TB disease treatment by combined SAT-DOT at 3% annual discount rate, Brazil and South Africa, 2020-2035 (US dollars in Millions) * 13](#_Toc104819133)

[**Table J.** Cumulative effectiveness projections at a 4% annual discount rate (in thousands), Brazil and South Africa, 2020–2035 ^*^ 14](#_Toc104819134)

[**Table K.** Cumulative undiscounted effectiveness projections (in thousands), Brazil and South Africa, 2020–2035 ^*^ 15](#_Toc104819135)

[**Fig A.** Incremental undiscounted costs, Brazil 16](#_Toc104819136)

[**Fig B.** Incremental costs discounted at a 4% annual rate, Brazil 16](#_Toc104819137)

[**Fig C.** Incremental undiscounted costs, South Africa 17](#_Toc104819138)

[**Fig D.** Incremental costs discounted at a 4% annual rate, South Africa 17](#_Toc104819139)

[**Fig E.** Threshold analysis of TPT, Brazil, Break-even point stratified by treatment group^*^ 18](#_Toc104819140)

[**Fig F.** Threshold analysis of TPT, South Africa, Break-even point stratified by treatment group^*^ 18](#_Toc104819141)

[**Fig G.** Varying the Rifampicin-resistance barrier, Brazil, (2020 – 2035) * 19](#_Toc104819142)

[**Fig H.** Rifampicin-resistance barrier, South Africa, (2020 – 2035) * 19](#_Toc104819143)

[**Fig I.** Varying the effect of TPT regimens on RR strains, Brazil * 20](#_Toc104819144)

[**Fig J.** The effect of TPT regimens on RR strains, South Africa * 20](#_Toc104819145)

[**Table L.** Cost estimates (95% UR) for TPT, and for TB disease treatment by universal DOT at 3% annual discount rate, Brazil and South Africa, 2020-2035 (US dollars in Millions) 21](#_Toc104819146)

[**Table M**. Incremental costs (95% UR) for TPT, and for TB disease treatment by universal DOT at 3% annual discount rate, Brazil and South Africa, 2020-2035 (US dollars in Millions) * 22](#_Toc104819147)

[**Fig K.** Incremental costs, 10-years scale up of TPT, South Africa 23](#_Toc104819148)

[**Fig L.** Incremental costs, 16-years scale up of TPT, South Africa 23](#_Toc104819149)

[**Fig M.** Incremental cost-effectiveness planes, 10-years scale up of TPT, South Africa ^*^ 24](#_Toc104819150)

[**Fig N.** Incremental cost-effectiveness planes, 16-years scale up of TPT, South Africa ^*^ 25](#_Toc104819151)

[**Table N.** Cumulative effectiveness projections with a 10-years scale up of TPT (in thousands), Brazil and South Africa, 2020–2035 ^*^ 26](#_Toc104819152)

[**Table O.** Cumulative effectiveness projections with a 16-years’ time horizon scale up of TPT (in thousands), Brazil and South Africa, 2020–2035 ^*^ 27](#_Toc104819153)

[**Fig O.** Incremental costs, 10-years scale up of TPT, Brazil 28](#_Toc104819154)

[**Fig P.** Incremental costs, 16-years scale up of TPT, Brazil 28](#_Toc104819155)

[**Fig Q.** Incremental cost-effectiveness planes, 10-years scale up of TPT, Brazil ^*^ 29](#_Toc104819156)

[**Fig R.** Incremental cost-effectiveness planes, 16-years scale up of TPT, Brazil ^*^ 30](#_Toc104819157)

##### **Table A**. Health system cost – programmatic management of TPT - Current Standard of Care

|  | Brazil | | | South Africa | | |
| --- | --- | --- | --- | --- | --- | --- |
| Parameter | **Value** | **Range** | **Reference** | **Value** |  | **Reference** |
| Cost of initial visit, total (nurse + MD) | $5.94 | ($2.96 - $11.86) | [1] | $1.66 |  | [2] |
| Cost/dose of 6H, dose (daily) | $0.02 |  | [3] | $0.02 |  | [3] |
| Number of Follow-up visits – 6H regimen | 6 |  |  | 6 |  |  |
| Number of Follow-up visits – minimal regimen | 3 |  |  | 3 |  |  |
| Number of Follow-up visits – optimal regimen | 1 |  |  | 1 |  |  |
| Cost of a single follow-up visit, (nurse + MD) | $5.94 | ($2.96 - $11.86) | [1] | $5.34 |  | [2] |
| 6H - standard diagnosis and treatment follow up (6-months) | **$39.49** |  |  | **$31.71** |  |  |
| Minimal regimen - diagnosis and treatment follow up (3-months) | **$24.85** |  |  | **$19.62** |  |  |
| Optimal regimen - diagnosis and treatment follow up (1-months) | **$15.79** |  |  | **$10.57** |  |  |

*Tuberculosis preventive treatment (TPT) ), Isoniazid (H), medical doctor (MD), six-months of daily isoniazid (6H)*

##### **Table B.** Regimens for TPT, DS-TB and RR-TB -Brazil

|  | Combination | Phase Duration (Months) | Daily/weekly cost | Total Cost | |
| --- | --- | --- | --- | --- | --- |
| TPT Regimen |  |  |  |  |  |
| 6H | INH | 6 | $0.02/day | $3.70 | **$3.70** |
| DS-TB Regimen |  |  |  |  |  |
| 2RHZE/4RH (6-months regimen) | 2RHZE (intensive phase) | 2 | $0.37/day | $18.13 | **$39.23** |
|  | 4RH (regular maintenance phase) | 4 | $0.22/day | $21.10 |  |
| RR-TB Regimen |  |  |  |  |  |
|  | 2S_5_ELTZ (1^st^ intensive phase) | 2 | $127.96/week | $1 109.38 | **$9 734.09** |
|  | 4S_3_ELTZ (2^nd^ intensive phase) | 4 | $126.37/week | $2 190.29 |  |
|  | 12ELT (regular continuation phase) | 12 | $123.74/week | $6 434.42 |  |

*Tuberculosis Preventive Treatment (TPT), Rifampicin-resistant (RR), drug-susceptible (DS), Pyrazinamide (Z), Ethambutol (E), Isoniazid (H),Streptomycin (S), Levofloxacin (L), Ethionamide (E) Terizidone (T), Linezolid (L), six-months of daily isoniazid (6H)*

##### **Table C.** Regimens for TPT, DS-TB and RR-TB – South Africa

|  | **Combination** | **Phase Duration (Months)** | **Daily cost** | **Total Cost *** |  |
| --- | --- | --- | --- | --- | --- |
| **TPT Regimen** |  |  |  |  |  |
| 6H | INH | 6 | 0.0206 | $3.82 | **$3.82** |
| **DS-TB Regimen** |  |  |  |  |  |
| 2RHZE/4RH (6-months regimen) | 2RHZE/4RH | 6 | 0 | $35.11 | **$35.11** |
| **RR - Long regimen** |  |  |  |  |  |
| Scenario 1: status quo | KM-MFX-ETO-TRD-Z | 18 | $7.16 | $3,912.39 | **$3,912.39** |
| Scenario 2: BDQ based regimen | LZD-BDQ_2wks_-LFX-CFZ-TRD | 0.5 | $16.75 | $254.06 | **$4,170.78** |
|  | LZD-BDQ_22wks_-LFX-CFZ-TRD | 5.5 | $9.85 | $1,643.37 |  |
|  | LFX-CFZ-TRD | 12 | $6.24 | $2,273.35 |  |
| Scenario 3: FLQ resistant or XDR *(INH & DLM)* | BDQ_2wks_-LZD-DLM-CFZ-TRD-Z-INH | 0.5 | $18.92 | $286.84 | **$4,629.81** |
|  | BDQ_22wks_-LZD-DLM-CFZ-TRD-Z-INH | 5.5 | $10.91 | $1,819.17 |  |
|  | LZD-CFZ-Z-INH-TRD | 12 | $6.94 | $2,523.79 |  |
| Scenario 4: FLQ resistant or XDR *(PAS & ETO)* | BDQ_2wks_-LZD-PAS-CFZ-TRD-Z-ETO | 0.5 | $19.82 | $300.61 | **$5,068.60** |
|  | BDQ_22wks_-LZD-PAS-CFZ-TRD-Z-ETO | 5.5 | $12.92 | $2,155.37 |  |
|  | LZD-CFZ-Z-ETO-TRD | 12 | $7.17 | $2,612.62 |  |
| **RR - Short regimen** |  |  |  |  |  |
| Scenario 1: with an injectable | KM-MFX-ETO-INH-CFZ-Z-E | 4 | $3.76 | $455.79 | **$677.52** |
|  | MFX-CFZ-Z-E | 5 | $1.47 | $221.73 |  |
| Scenario 2: injectable-free | LZD_2months_-BDQ_2wks_-LFX-CFZ_2months_-INH-Z-E | 0.5 | $11.60 | $175.93 | **$727.18** |
|  | LZD_2months_-BDQ_6wks_-LFX-CFZ_2months_-INH-Z-E | 1.5 | $4.70 | $213.78 |  |
|  | BDQ-LFX-CFZ-INH-Z-E | 2 | $2.97 | $180.52 |  |
|  | LFX-CFZ-Z-E | 5 | $1.03 | $156.96 |  |

** (Total calculation is done by detailing doses per week for the entire duration of treatment in months)* **,** *Tuberculosis Preventive Treatment (TPT), rifampicin-resistant (RR), drug-susceptible (DS), Kanamycin (Km), Levofloxacin (Lfx), Clofazimine (CFZ) , Pyrazinamide (Z), Ethambutol (E), Isoniazid (INH), Ethionamide (Eto), Moxifloxacin (Mfx), Terizidone (Trd), p-aminosalicylic acida (PAS), Bedaquiline (Bdq), Linezolid (LZD), Delamanid (Dim), Rifabutin (Rbt), six-months of daily isoniazid (6H)*

##### **Table D**. Health system cost – drug-susceptible TB disease diagnosis and treatment - Current Standard of Care

|  | Value | Range or SD | Reference | Value | Range or SD | Reference | |
| --- | --- | --- | --- | --- | --- | --- | --- |
|  | Brazil | | | South Africa | | |  |
| TB Pre-Diagnosis |  |  |  |  |  |  | |
| Cost of medical Consultation: initial assessment | **$16.70** | **($8.36 - $33.41)** | [4] | **$1.71** |  | [2] | |
| Cost of Complementary exams | **$3.87** | **($12.87)** | [5] | **$13.73** |  | [6] | |
| Course of antibiotics |  |  |  | ***$0.38*** | *($0.11)* | *[7]* | |
| *Subtotal - Pre-Diagnosis* | ***$20.57*** |  |  | ***$15.82*** |  |  | |
| Standard Initial tests for diagnosis |  |  |  |  |  |  | |
| Cost per CXR | **$15.80** | **($7.89 - $31.61)** | [4] | **$19.67** |  | [8] | |
| Cost per Xpert MTB/RIF | **$16.70** | **($8.36 - $33.41)** | [4] | **$40.71** |  | [6] | |
| Proportion who had culture done |  |  | [9] |  |  | [10] | |
| Prorated cost of Culture (for Xpert negative high suspicion) ^υ^ | **$1.37** |  | [4] | **$4.06** |  | [7] | |
| *Total Diagnostic cost* | ***$33.77*** |  |  | ***$64.44*** |  |  | |
| Treatment and Follow up |  |  |  |  |  |  | |
| Fixed-dose combination 2RHZE/4RH (6-months regimen) | **$35.11** |  | [3] | **$36.22** |  | [3] | |
| Pro-rated (For Brazil -Hospitalization for 24 days (pro-rated at 5.19 per day per TB case)) | **$139.46** |  | [11] | **$6.45** |  | [12] | |
| Cost per DOT visit | **$9.45** |  | [5] | **$3.12** |  | [2] | |
| *Subtotal - DOT visits (regular treatment)* | ***$573.35*** |  | [4, 13]*** | **$411.69** |  | [2] | |
| Mixed model -Proportion of patients under DOT | **65%** |  | [14] | **20%** |  | [2] | |
| Cost per Follow-up visit with medical doctor | **$16.63** | **($8.32 - $33.25)** | [4] | **$5.15** |  | [2, 15] | |
| *Subtotal - Follow-up visit (regular treatment: 6 visits)* | ***$99.75*** |  |  | ***$30.90*** |  |  | |
| Cost per sputum smear (per sample) | **$3.50** | **($0.99 - $6.97)** | [4] | **$13.73** |  | [6] | |
| Cost per Liver Function Test | **$4.24** | **($2.11 - $8.39)** | [1] | **$19.00** |  | [12] | |
| Line probe assay (LPA) or drug susceptibility testing (DST) – South Africa |  |  |  | **$115.52** |  | *[15, 16]* | |
| Proportion: special circumstances (CLD, not responding to treatment, etc.) |  |  |  | ***19%*** |  | *[15]* | |
| *Subtotal - Follow-up lab/tests (regular treatment: 3 smears, DST and 1 LFT)* | ***$14.73*** |  |  | ***$39.29*** |  |  | |
| CXR, during treatment (2 done) | **$31.61** |  | [4] | **$39.34** |  | [8, 15] | |
| Proportion of previously treated individuals as a proxy of those with complications that will require a 9-month treatment |  |  |  | ***7.10%*** |  | *[15]* | |
| Proportion of previously treated individuals as a proxy of those with complications that will require a 9-month treatment |  |  |  | ***7.10%*** |  | *[15]* | |
| *Treatment and Follow up – DOT model (Regular 6-months treatment)*  *Treatment and Follow up –* *Mixed SAT/DOT model* | ***$894.00***  ***$694.36*** |  |  | ***$541.19***  ***$200.16*** |  |  | |
| Grand total – pre-diagnosis, standard diagnosis and treatment follow up (DOT model)  Grand total – pre-diagnosis, standard diagnosis and treatment follow up (Mixed SAT/DOT) | ***$948***  ***$747*** |  |  | ***$621***  ***$280*** |  |  | |

*Standard deviation (SD), tuberculosis (TB), chest x-ray (CXR), rifampicin (R), isoniazid (H), pyrazinamide (Z), ethambutol (E), directly observed therapy (DOT), liver function test (LFT), self administered therapy (SAT), chronic liver disease (CLD)*

*^υ^ from Brazilian TB guidelines, culture is only done when there is high clinical suspicion but Xpert result is negative, for South Africa – guidelines stipulate that, a culture is done when high clinical suspicion but negative test result (we are using a complement of sensitivity of X-pert)*

***Pre-diagnostic and Diagnosis costs applied only to incident active cases or relapse cases who are subsequently retreated. Treatment and follow up costs applied to all active cases.*

**** Calculated inputs from listed publications*

##### **Table E**. Health system cost -- RR-TB disease treatment - Current standard of care

|  | Brazil | | | South Africa | | |
| --- | --- | --- | --- | --- | --- | --- |
| Parameter | **Value** | **Range** | **Reference** | **Value** | **Range** | **Reference** |
| *Sub-total for pre-diagnosis and initial diagnostic testing (standard +additional)*  *(i.e. $20.57 + $33.77 for Brazil & $15.82 + $64.44 for South Africa* | *$54.34* |  | *table D above* | *$80.26* |  | *table D above* |
| Treatment and Follow up |  |  |  |  |  |  |
| Fixed-dose combination: 2S_5_ELTZ (18-months regimen) | **$8,709.24** |  | [3] |  |  |  |
| Hospitalization for 24 days (prorated per TB case) | **$139.46** |  | [11] |  |  |  |
| DOT visits (regular treatment: 182 visits) | **$1,720.04** |  | [4, 13]* |  |  |  |
| Follow-up consultation (regular treatment: 18 visits) | **$299.23** |  | [4] |  |  |  |
| Cost per Culture | **$9.36** | ($4.67 - $18.71) | [4] |  |  |  |
| Cost for a complete blood count (CBC) | **$2.89** | ($1.43 - $5.77) | [1] |  |  |  |
| *Total cost – regular 18-months treatment* | ***$11,146.00*** |  |  |  |  |  |

*Streptomycin (S), Ethambutol (E), Linezolid (L), Terizidone (T), Pyrazinamide (Z), Tuberculosis (TB), Directly observed therapy (DOT)*

** Calculated inputs from given publications, Brazil Follow-up lab/tests (regular treatment: 18 smears, 6 cultures, 9 CBC and 9 LFT, 6CXR)*

##### **Table F.** Epidemiological estimates (95% uncertainty ranges, UR) (in thousands) at 3% annual discount rate, Brazil and South Africa, 2020–2035

|  | Status quo | Scale up 6H | Minimal TPT | Optimal TPT |
| --- | --- | --- | --- | --- |
| Brazil | | | | |
| All treatment candidates | | | | |
| No. of people initiating TPT | **669**  (507, 810) | **3 721**  (3 301, 4 009) | **3 712**  (3 296, 3 998) | 3 694  (3 288, 3 973) |
| TB cases (DS-TB & RR-TB) | **1 128**  (1052, 1 199) | **1 124**  (1 053,1 189) | **1 095**  (1 027,1 161) | 1 039  (978,1 096) |
| DS-TB cases | **1 093**  (1 017, 1 165) | **1 089**  (1 019, 1 158) | **1 059**  (989, 1 124) | 1 005  (945, 1 063) |
| RR-TB cases | **35**  (25, 49) | **35**  (25, 49) | **36**  (26, 50) | 33  (24, 47) |
| Deaths | **123**  (115, 132) | **123**  (115, 131) | **120**  (112, 128) | 113  (107, 120) |
| TB DALYs | **2 542**  (2 101, 3 118) | **2 528**  (2 087, 3 101) | **2 479**  (2 044, 3 044) | 2 313  (1 913, 2 859) |
| Household contacts (HIV negative) | | | | |
| No. of people initiating TPT | **0** | **2 610**  (2 366, 2 837) | **2 601**  (2 362, 2 827) | 2 583  (2 354, 2 800) |
| TB cases (DS-TB & RR-TB) | **979**  (910, 1 046) | **972**  (907, 1 033) | **957**  (891, 1 015) | 931  (870, 982) |
| DS-TB cases | **949**  (877, 1 016) | **942**  (875, 1 005) | **926**  (862, 985) | 901  (841, 955) |
| RR-TB cases | **31**  (22, 43) | **31**  (22, 43) | **31**  (22, 44) | 29  (21, 41) |
| Deaths | **98**  (93, 104) | **98**  (93, 103) | **96**  (92, 101) | 94  (90, 98) |
| TB DALYs | **1 904**  (1 588, 2 312) | **1 891**  (1 582, 2 301) | **1 867**  (1 568, 2 277) | 1 815  (1 518, 2 234) |
| PLHIV | | | | |
| No. of people initiating TPT | **669**  (507, 810) | **1 110**  (912, 1225) | **1 111**  (912, 1225) | 1 111  (913, 1225) |
| TB cases (DS-TB & RR-TB) | **149**  (129, 173) | **152**  (133, 174) | **138**  (119, 162) | 108  (91, 128) |
| DS-TB cases | **145**  (125, 168) | **147**  (128, 169) | **133**  (114, 156) | 104  (88, 123) |
| RR-TB cases | **5**  (3, 7) | **5**  (3, 7) | **5**  (3, 8) | 4  (3, 6) |
| Deaths | **25**  (19, 31) | **25**  (20, 31) | **24**  (18, 30) | 19  (15, 25) |
| TB DALYs | **638**  (427, 883) | **637**  (428, 875) | **612**  (410, 839) | 498  (330, 698) |
| South Africa | | | | |
| All treatment candidates | | | | |
| No. of people initiating TPT | **4 109**  (3 252, 4 852) | **10 135**  (8 521, 11 417) | **10 071**  (8 516, 11 338) | 9 920  (8 454, 11 049) |
| TB cases (DS-TB & RR-TB) | **3 663**  (3 002, 4 393) | **3 398**  (2 800, 4 023) | **3 015**  (2 477, 3 600) | 2 164  (1 826, 2 517) |
| DS-TB cases | **3 479**  (2 805, 4 243) | **3 223**  (2 644, 3 857) | **2 818**  (2 282, 3 418) | 2 017  (1 675, 2 379) |
| RR-TB cases | **184**  (98, 316) | **175**  (93, 298) | **197**  (109, 329) | 147  (77, 253) |
| Deaths | **843**  (548, 1 201) | **788**  (518, 1 122) | **718**  (470, 1 034) | 531  (359, 752) |
| TB DALYs | **36 682**  (19 459, 58 024) | **34 159**  (17 903, 54 184) | **31 067**  (15 946, 49 928) | 22 736  (11 995, 35 847) |
| Household contacts (HIV negative) | | | | |
| No. of people initiating TPT | **0** | **5 196**  (4 436, 5 818) | **5 109**  (4 370, 5 732) | 4 903  (4 261, 5 562) |
| TB cases (DS-TB & RR-TB) | **1 480**  (1 023, 1 959) | **1 307**  (918, 1 698) | **1 192**  (861, 1 522) | 931  (692, 1 161) |
| DS-TB cases | **1 398**  (958, 1 879) | **1 231**  (859, 1 616) | **1 109**  (787, 1 441) | 868  (637, 1 105) |
| RR-TB cases | **82**  (46, 138) | **76**  (42, 126) | **83**  (46, 138) | 63  (34, 106) |
| Deaths | **257**  (230, 283) | **230**  (208, 252) | **214**  (194, 235) | 173  (158, 190) |
| TB DALYs | **10 425**  (7 796, 13 103) | **9 316**  (6 898, 11 856) | **8 603**  (6 264, 11 113) | 6 886  (4 930, 9 304) |
| PLHIV | | | | |
| No. of people initiating TPT | **4 109**  (3 252, 4 852) | **4 939**  (4 044, 5 714) | **4 962**  (4 060, 5 726) | 5 017  (4 109, 5 776) |
| TB cases (DS-TB & RR-TB) | **2 183**  (1 569, 2 865) | **2 091**  (1 514, 2 706) | **1 822**  (1 273, 2 453) | 1 232  (873, 1 616) |
| DS-TB cases | **2 085**  (1 478, 2 724) | **2 000**  (1 428, 2 576) | **1 706**  (1 180, 2 307) | 1 145  (818, 1 521) |
| RR-TB cases | **96**  (49, 189) | **93**  (48, 183) | **108**  (55, 205) | 78  (39, 156) |
| Deaths | **586**  (314, 939) | **557**  (307, 887) | **504**  (270, 817) | 358  (193, 569) |
| TB DALYs | 26 257  (11 087, 46 706) | 24 843  (10 701, 43 873) | 22 464  (9 442, 40 332) | 15 850  (6 655, 28 171) |

*Uncertainty ranges (UR), six-months of daily isoniazid (6H), Tuberculosis preventive treatment (TPT), drug-susceptible tuberculosis (DS-TB), rifampicin-resistant tuberculosis (RR-TB), disability-adjusted life years (DALYs), persons living with HIV (PLHIV)*

##### **Table G.** Incremental effectiveness (in thousands) at 3% annual discount rate, Brazil and South Africa, 2020-2035

|  | **6H vs Status quo ^*^** | **Minimal vs status quo** | **Minimal vs 6H** | **Optimal vs 6H** | **Optimal vs minimal** |
| --- | --- | --- | --- | --- | --- |
| **Brazil** | | | | | |
| **All treatment candidates** | | | | | |
| 1. No. of people initiating TPT | **3 051**  (2 753, 3 332) | **3 043**  (2 746, 3 319) | **-8**  (-12, -4) | -27  (-41, -12) | **-18**  (-30, -8) |
| 2. DS-TB cases | **-5**  (-13, 7) | **-35**  (-44, -25) | **-30**  (-36, -25) | **-83**  (-100, -66) | **-53**  (-68, -39) |
| 3. RR-TB cases | **-0.07**  (-0.27, 0.19) | **0.90**  (0.71, 1.14) | **0.96**  (0.74, 1.27) | **-2**  (-3, -1) | **-3**  (-4, -2) |
| 4. All TB cases (DS-TB & RR-TB) | **-5**  (-13, 7) | **-34**  (-43, -24) | **-29**  (-35, -24) | **-85**  (-102, -67) | **-56**  (-71, -41) |
| 5. Deaths | **-1**  (-1, 1) | **-3**  (-4, -2) | **-3**  (-3, -2) | **-10**  (-12, -8) | **-7**  (-9, -5) |
| 6. TB DALYs | **-14**  (-33, 11) | **-63**  (-84, -42) | **-49**  (-66, -36) | **-215**  (-282, -152) | **-165**  (-229, -106) |
| **Household contacts (HIV negative)** | | | | | |
| 1. No. of people initiating TPT | **2 610**  (2 366, 2 837) | **2 601**  (2 362, 2 826) | **-9**  (-12, -5) | **-28**  (-43, -13) | **-19**  (-30, -8) |
| 2. DS-TB cases | **-7**  (-13, 1) | **-23**  (-32, -13) | **-16**  (-21, -11) | **-40**  (-55, -25) | **-24**  (-35, -14) |
| 3. RR-TB cases | **-0.13**  (-0.32, 0.07) | **0.34**  (0.20, 0.51) | **0.48**  (0.29, 0.70) | **-1**  (-2, -1) | **-2**  (-2, -1) |
| 4. All TB cases (DS-TB & RR-TB) | **-7**  (-14, 1) | **-23**  (-32, -13) | **-16**  (-21, -10) | **-41**  (-57, -26) | **-26**  (-37, -15) |
| 5. Deaths | **-1**  (-1, 0) | **-2**  (-3, -1) | **-1**  (-2, -1) | **-4**  (-5, -2) | **-2**  (-3, -1) |
| 6. TB DALYs | **-13**  (-25, 2) | **-36**  (-53, -20) | **-24**  (-32, -15) | **-76**  (-102, -46) | **-52**  (-73, -31) |
| **PLHIV** | | | | | |
| 1. TPT | **441**  (314, 562) | **442**  (314, 562) | 0.43  (0.19, 0.69) | **1**  (0.35, 2) | **1**  (0, 1) |
| 2. DS-TB cases | **2**  (-0.22, 6) | **-12**  (-14, -10) | -14  (-18, -11) | **-43**  (-48, -37) | **-29**  (-35, -23) |
| 3. RR-TB cases | **0.06**  (0.02, 0.14) | 0.55  (0.45, 0.65) | 0.49  (0.40, 0.58) | **-0.55**  (-0.84, 0.34) | **-1**  (-1, -1) |
| 4. All TB cases (DS-TB & RR-TB) | **2**  (-0.19, 6) | **-11**  (-13, -9) | -13  (-17, -11) | **-44**  (-49, -37) | **-30**  (-36, -23) |
| 5. Deaths | **0.13**  (-0.21, 1) | **-1**  (-2, -1) | -1  (-2, -1) | **-6**  (-7, -5) | **-5**  (-6, -3) |
| 6. TB DALYs | **-1**  (-10, 9) | **-27**  (-38, -17) | -25  (-41, -15) | **-139**  (-193, -91) | **-113**  (-164, -69) |
| **South Africa** | | | | | |
| **All treatment candidates** | | | | | |
| 1. TPT | **6 026**  (5 020, 7 031) | **5 962**  (4 991, 6 943) | **-64**  (-153, -6) | **-215**  (-572, -18) | **-150**  (-409, -12) |
| 2. DS-TB cases | **-256**  (-395, -128) | **-661**  (-870, -492) | **-405**  (-515, -306) | **-1 206**  (-1 532, -923) | **-801**  (-1 074, -596) |
| 3. RR-TB cases | **-9**  (-18, -4) | **13**  (4, 22) | **22**  (11, 37) | **-28**  (-48, -16) | **-50**  (-82, -29) |
| 4. All TB cases (DS-TB & RR-TB) | **-265**  (-404, -137) | **-648**  (-861, -475) | **-384**  (-494, -282) | **-1 234**  (-1 567, -945) | **-851**  (-1 121, -641) |
| 5. Deaths | **-55**  (-91, -27) | **-124**  (-178, -82) | **-69**  (-94, -46) | **-257**  (-379, -159) | **-187**  (-291, -107) |
| 6. TB DALYs | **-2 523**  (-4 354, -1 187) | **-5 615**  (-8 471, -3 146) | **-3 092**  (-4 490, -1 825) | **-11 423**  (-18 285, -5 830) | **-8 331**  (-14 084, -3 960) |
| **Household contacts (HIV negative)** | | | | | |
| 1. TPT | **5 196**  (4 436, 5 818) | **5 109**  (4 370, 5 732) | **-87**  (-170, -27) | **-293**  (-626, -83) | **-206**  (-468, -57) |
| 2. DS-TB cases | **-166**  (-253, -95) | **-288**  (-436, -163) | **-122**  (-190, -64) | **-363**  (-531, -211) | **-241**  (-341, -148) |
| 3. RR-TB cases | **-6**  (-11, -3) | **1**  (-1, 4) | **7**  (4, 12) | **-13**  (-21, -7) | **-20**  (-32, -11) |
| 4. All TB cases (DS-TB & RR-TB) | **-172**  (-260, -99) | **-287**  (-436, -161) | **-115**  (-184, -59) | **-376**  (-546, -220) | **-261**  (-366, -164) |
| 5. Deaths | **-26**  (-35, -19) | **-42**  (-54, -32) | **-16**  (-22, -11) | **-58**  (-71, -45) | **-42**  (-51, -33) |
| 6. TB DALYs | **-1 109**  (-1 440, -782) | **-1 822**  (-2 221, -1 396) | **-713**  (-895, -531) | **-2 430**  (-3 041, -1 815) | **-1 717**  (-2 198, -1 262) |
| **PLHIV** | | | | | |
| 1. TPT | **830**  (286, 1 441) | **853**  (307, 1 470) | **23**  (8, 40) | **78**  (40, 138) | **55**  (25, 98) |
| 2. DS-TB cases | **-89**  (-169, -17) | **-373**  (-481, -274) | **-283**  (-342, -211) | **-843**  (-1 120, -608) | **-559**  (-790, -375) |
| 3. RR-TB cases | **-3**  (-7, -1) | **11**  (5, 20) | **15**  (7, 26) | **-16**  (-28, -8) | **-30**  (-51, -16) |
| 4. All TB cases (DS-TB & RR-TB) | **-93**  (-172, -20) | **-361**  (-467, -267) | **-269**  (-327, -201) | **-858**  (-1 131, -621) | **-590**  (-826, -400) |
| 5. Deaths | **-29**  (-59, -7) | **-82**  (-129, -45) | **-53**  (-76, -33) | **-199**  (-314, -105) | **-146**  (-246, -70) |
| 6. TB DALYs | **-1 414**  (-2 971, -330) | **-3 793**  (-6 392, -1 674) | **-2 379**  (-3 698, -1 203) | **-8 993**  (-15 500, -3 861) | **-6 614**  (-12 032, -2 593) |

** negative values indicate cases, deaths and DALYs averted*

*Uncertainty ranges (UR), six-months of daily isoniazid (6H), Tuberculosis preventive treatment (TPT), drug-susceptible tuberculosis (DS-TB), rifampicin-resistant tuberculosis (RR-TB), disability-adjusted life years (DALYs), persons living with HIV (PLHIV)*

##### **Table H.** Cost estimates (95% UR) for TPT, and for TB disease treatment by combined SAT-DOT at 3% annual discount rate, Brazil and South Africa, 2020-2035 (US dollars in Millions)

| **Cost (95% UR) of TPT and TB management by SAT-DOT (in US dollars in Millions), 2020-2035** | | | | |
| --- | --- | --- | --- | --- |
| **Cost** | **Status quo** | **Scale-up 6H** | **Minimal - TPT** | **Optimal - TPT** |
| **Brazil** | | | | |
| TPT | **$25**  ($19, $30) | **$134**  ($118, $148) | **$90**  ($79, $99) | **$62**  ($55, $69) |
| Diagnosis and treatment of DS-TB | **$768**  ($697, $841) | **$765**  ($696, $834) | **$746**  ($678, $814) | **$711**  ($647, $773) |
| Diagnosis and treatment of RR-TB | **$348**  ($242, $482) | **$347**  ($242, $481) | **$356**  ($249, $492) | **$331**  ($231, $459) |
| Diagnosis and treatment of TB disease (DS-TB+RR-TB) | **$1 116**  ($986, $1 265) | **$1 112**  ($990, $1 256) | **$1 102**  ($979, $1 249) | **$1 042**  ($933, $1 177) |
| Total cost (TPT & TB) | **$1 140**  ($1 009, $1 291) | **$1 247**  ($1 119, $1 400) | **$1 191**  ($1 064, $1 346) | **$1 104**  ($991, $1 243) |
| **South Africa** | | | | |
| TPT | **$145**  ($113, $172) | **$349**  ($290, $401) | **$248**  ($207, $285) | **$172**  ($144, $196) |
| Diagnosis and treatment of DS-TB | **$910**  ($734, $1 115) | **$850**  ($692, $1 029) | **$751**  ($602, $915) | **$553**  ($453, $655) |
| Diagnosis and treatment of RR-TB | **$1 480**  ($799, $2 533) | **$1 415**  ($762, $2 390) | **$1 577**  ($866, $2 637) | **$1 203**  ($634, $2 066) |
| Diagnosis and treatment of TB disease (DS-TB+RR-TB) | **$2 391**  ($1 656, $3 414) | **$2 265**  ($1 575, $3 233) | **$2 328**  ($1 588, $3 356) | **$1 755**  ($1 182, $2 614) |
| Total cost (TPT & TB) | **$2 535**  ($1 801, $3 560) | **$2 614**  ($1 922, $3 580) | **$2 576**  ($1 840, $3 624) | **$1 927**  ($1 353, $2 786) |

*Uncertainty ranges (UR), Tuberculosis preventive treatment (TPT), combined self-administered and directly observed treatment (SAT-DOT), six-months of daily isoniazid (6H), tuberculosis (TB), persons living with HIV (PLHIV), drug-susceptible tuberculosis (DS-TB), rifampicin-resistant tuberculosis (RR-TB)*

##### **Table I**. Incremental costs (95% UR) for TPT, and for TB disease treatment by combined SAT-DOT at 3% annual discount rate, Brazil and South Africa, 2020-2035 (US dollars in Millions) *

|  | **6H vs Status quo ^**^** | **Minimal vs Status quo** | **Minimal vs 6H** | **Optimal vs 6H** | **Optimal vs minimal** |
| --- | --- | --- | --- | --- | --- |
| **Brazil** | | | | | |
| **All treatment candidates** | | | | | |
| Cost of TPT | **$109**  ($97, $122) | **$65**  ($57, $73) | **-$45**  (-$55, -$34) | **-$72**  (-$82, -$61) | **-$27**  (-$33, -$21) |
| Cost of diagnosis and treatment of TB disease | **-$3**  (-$10, $6) | **-$14**  (-$21, -$7) | **-$11**  (-$15, -$6) | **-$70**  (-$86, -$55) | **-$60**  (-$75, -$44) |
| Total cost (TPT & TB) | **$106**  ($95, $117) | **$51**  ($41, $60) | **-$55**  (-$65, -$45) | **-$142**  (-$163, -$120) | **-$87**  (-$105, -$69) |
| **Household contacts (HIV negative)** | | | | | |
| Cost of TPT | **$93**  ($83, $103) | **$62**  ($56, $69) | **-$31**  (-$38, -$24) | **-$50**  (-$57, -$43) | **-$19**  (-$23, -$15) |
| Cost of diagnosis and treatment of TB disease | **-$6**  (-$11, $1) | **-$12**  (-$18, -$5) | **-$6**  (-$9, -$3) | **-$37**  (-$49, -$23) | **-$31**  (-$42, -$18) |
| Total cost (TPT & TB) | **$87**  ($78, $97) | **$50**  ($42, $59) | **-$37**  (-$44, -$30) | **-$87**  (-$101, -$72) | **-$50**  (-$62, -$37) |
| **PLHIV** | | | | | |
| Cost of TPT | **$16**  ($11, $21) | **$3**  (-$2, $7) | **-$14**  (-$17, -$10) | **-$22**  (-$26, -$18) | **-$8**  (-$11, -$6) |
| Cost of diagnosis and treatment of TB disease | **$2**  ($0, $6) | **-$2**  (-$4, -$1) | **-$5**  (-$7, -$3) | **-$34**  (-$39, -$28) | **-$29**  (-$35, -$23) |
| Total cost (TPT & TB) | **$19**  ($13, $24) | **$1**  (-$4, $6) | **-$18**  (-$22, -$14) | **-$56**  (-$63, -$46) | **-$38**  (-$44, -$30) |
| **South Africa** | | | | | |
| **All treatment candidates** | | | | | |
| Cost of TPT | **$204**  ($168, $243) | **$103**  ($76, $132) | **-$102**  (-$129, -$77) | **-$178**  (-$213, -$143) | **-$76**  (-$95, -$57) |
| Cost of diagnosis and treatment of TB disease | **-$126**  (-$199, -$74) | **-$62**  (-$143, $26) | $63  (-$9, $171) | **-$509**  (-$671, -$386) | **-$573**  (-$830, -$392) |
| Total cost (TPT & TB) | **$79**  (-$7, $151) | **$41**  (-$52, $140) | **-$38**  (-$109, $70) | **-$687**  (-$860, -$548) | **-$649**  (-$903, -$457) |
| **Household contacts (HIV negative)** | | | | | |
| Cost of TPT | **$175**  ($147, $200) | **$122**  ($103, $140) | **-$52**  (-$65, -$40) | **-$92**  (-$110, -$74) | **-$40**  (-$50, -$30) |
| Cost of diagnosis and treatment of TB disease | **-$83**  (-$130, -$49) | **-$60**  (-$105, -$25) | **$22**  (-$7, $58) | **-$183**  (-$273, -$116) | **-$206**  (-$313, -$133) |
| Total cost (TPT & TB) | **$92**  ($32, $138) | **$62**  ($5, $105) | **-$30**  (-$59, $8) | **-$275**  (-$359, -$210) | **-$245**  (-$349, -$173) |
| **PLHIV** | | | | | |
| Cost of TPT | **$30**  ($11, $52) | **-$19**  (-$41, $2) | **-$49**  (-$64, -$36) | **-$86**  (-$105, -$67) | **-$36**  (-$46, -$27) |
| Cost of diagnosis and treatment of TB disease | **-$43**  (-$80, -$10) | **-$2**  (-$43, $52) | **$41**  (-$3, $117) | **-$326**  (-$443, -$226) | **-$367**  (-$554, -$223) |
| Total cost (TPT & TB) | **-$13**  (-$63, $33) | **-$21**  (-$71, $43) | **-$8**  (-$53, $68) | **-$412**  (-$537, -$300) | **-$404**  (-$588, -$258) |

** TB management is done by a mixed model of SAT-DOT ** negative values indicate cost savings*

*Uncertainty ranges (UR), six-months of daily isoniazid (6H), Tuberculosis preventive treatment (TPT) ), six-months of daily isoniazid (6H), tuberculosis (TB), persons living with HIV (PLHIV)*

##### **Table J.** Cumulative effectiveness projections at a 4% annual discount rate (in thousands), Brazil and South Africa, 2020–2035 ^*^

|  | Status quo | Scale up 6H | Minimal TPT | Optimal TPT |
| --- | --- | --- | --- | --- |
| Brazil | | | | |
| 1. No. of people initiating TPT | **628**  (475, 761) | **3 459**  (3 066, 3 727) | **3 452**  (3 062, 3 718) | 3 435  (3 055, 3 696) |
| 2. DS-TB cases | **1 057**  (986, 1 123) | **1 053**  (987, 1 114) | **1 027**  (964, 1 088) | 975  (919, 1 029) |
| 3. RR-TB cases | **33**  (23, 46) | **33**  (23, 46) | **34**  (24, 47) | 31  (22, 44) |
| 4. All TB cases (DS-TB & RR-TB) | **1 090**  (1 052, 1 199) | **1 086**  (1 053, 1 189) | **1 061**  (964, 1 150) | 1 006  (978, 1 096) |
| 5. Deaths | **116**  (108, 124) | **115**  (108, 123) | **113**  (105, 120) | 106  (100, 112) |
| 6. TB DALYs | **2 381**  (1 968, 2 921) | **2 369**  (1 955, 2 904) | **2 324**  (1 917, 2 856) | 2 172  (1 796, 2 683) |
| South Africa | | | | |
| 1. No. of people initiating TPT | **3 861**  (3 050, 4 564) | **9 455**  (7 935, 10 664) | **9 396**  (7 930, 10 586) | 9 258  (7 869, 10 327) |
| 2. DS-TB cases | **3 262**  (2 637, 3 972) | **3 030**  (2 485, 3 622) | **2 658**  (2 151, 3 214) | 1 921  (1 594, 2 263) |
| 3. RR-TB cases | **172**  (92, 294) | **164**  (88, 278) | **183**  (102, 305) | 138  (73, 237) |
| 4. All TB cases (DS-TB & RR-TB) | **3 434**  (2 818, 4 116) | **3 193**  (2 637, 3 774) | **2 842**  (2 333, 3 391) | 2 059  (1 735, 2 393) |
| 5. Deaths | **790**  (514, 1 127) | **740**  (487, 1 054) | **677**  (443, 974) | 505  (341, 714) |
| 6. TB DALYs | 34 393  (18 204, 54 233) | 32 104  (16 794, 50 797) | 29 282  (15 044, 47 027) | 21 637  (11 378, 34 109) |

** The discount factor applied was 1/(1+D)^t^, where D is the 4% discount rate that is applied to each period t=year, TB disease management by a mixed model of SAT-DOT*

*Uncertainty ranges (UR), Tuberculosis preventive treatment (TPT), six-months of daily isoniazid (6H), drug-susceptible tuberculosis (DS-TB), rifampicin-resistant tuberculosis (RR-TB), disability-adjusted life years (DALYs), persons living with HIV (PLHIV)*

##### **Table K.** Cumulative undiscounted effectiveness projections (in thousands), Brazil and South Africa, 2020–2035 ^*^

|  | Status quo | Scale up 6H | Minimal TPT | Optimal TPT |
| --- | --- | --- | --- | --- |
| Brazil | | | | |
| 1. No. of people initiating TPT | **821**  (624, 991) | **4 697**  (4 180, 5 061) | **4 686**  (4 173, 5 046) | 4 661  (4 160, 5 009) |
| 2. DS-TB cases | **1 350**  (1 254, 1 440) | **1 343**  (1 256, 1 430) | **1 304**  (1 216, 1 386) | 1 234  (1 161, 1 306) |
| 3. RR-TB cases | **44**  (31, 62) | **44**  (31, 61) | **45**  (32, 63) | 41  (29, 58) |
| 4. All TB cases (DS-TB & RR-TB) | **1 394**  (1 296, 1 482) | **1 387**  (1 299, 1 468) | **1 348**  (1 263, 1 430) | 1 275  (1 200, 1 346) |
| 5. Deaths | **152**  (142, 163) | **152**  (142, 162) | **148**  (138, 158) | 139  (131, 147) |
| 6. TB DALYs | **3 142**  (2 598, 3 856) | **3 123**  (2 578, 3 838) | **3 056**  (2 521, 3 751) | 2 838  (2 351, 3 509) |
| South Africa | | | | |
| 1. No. of people initiating TPT | **5 032**  (4 006, 5 916) | **12 670**  (10 715, 14 193) | **12 585**  (10 709, 14 092) | 12 390  (10 641, 13 742) |
| 2. DS-TB cases | **4 289**  (3 471, 5 255) | **3 942**  (3 216, 4 734) | **3 409**  (2 757, 4 150) | 2 370  (1 969, 2 792) |
| 3. RR-TB cases | **230**  (122, 397) | **217**  (115, 372) | **246**  (136, 414) | 179  (93, 314) |
| 4. All TB cases (DS-TB & RR-TB) | **4 518**  (3 688, 5 445) | **4 160**  (3 425, 4 936) | **3 655**  (2 988, 4 381) | 2 549  (2 157, 2 967) |
| 5. Deaths | **1 039**  (673, 1 473) | **964**  (632, 1 370) | **872**  (565, 1 253) | 626  (423, 891) |
| 6. TB DALYs | 45 227  (24 132, 71 906) | 41 806  (22 041, 66 291) | 37 686  (19 427, 60 535) | 26 761  (14 210, 42 211) |

** TB disease management by a mixed model of SAT-DOT*

*Uncertainty ranges (UR), Tuberculosis preventive treatment (TPT), six-months of daily isoniazid (6H), drug-susceptible tuberculosis (DS-TB), rifampicin-resistant tuberculosis (RR-TB), disability-adjusted life years (DALYs), persons living with HIV (PLHIV)*

##### **Fig A.** Incremental undiscounted costs, Brazil

***
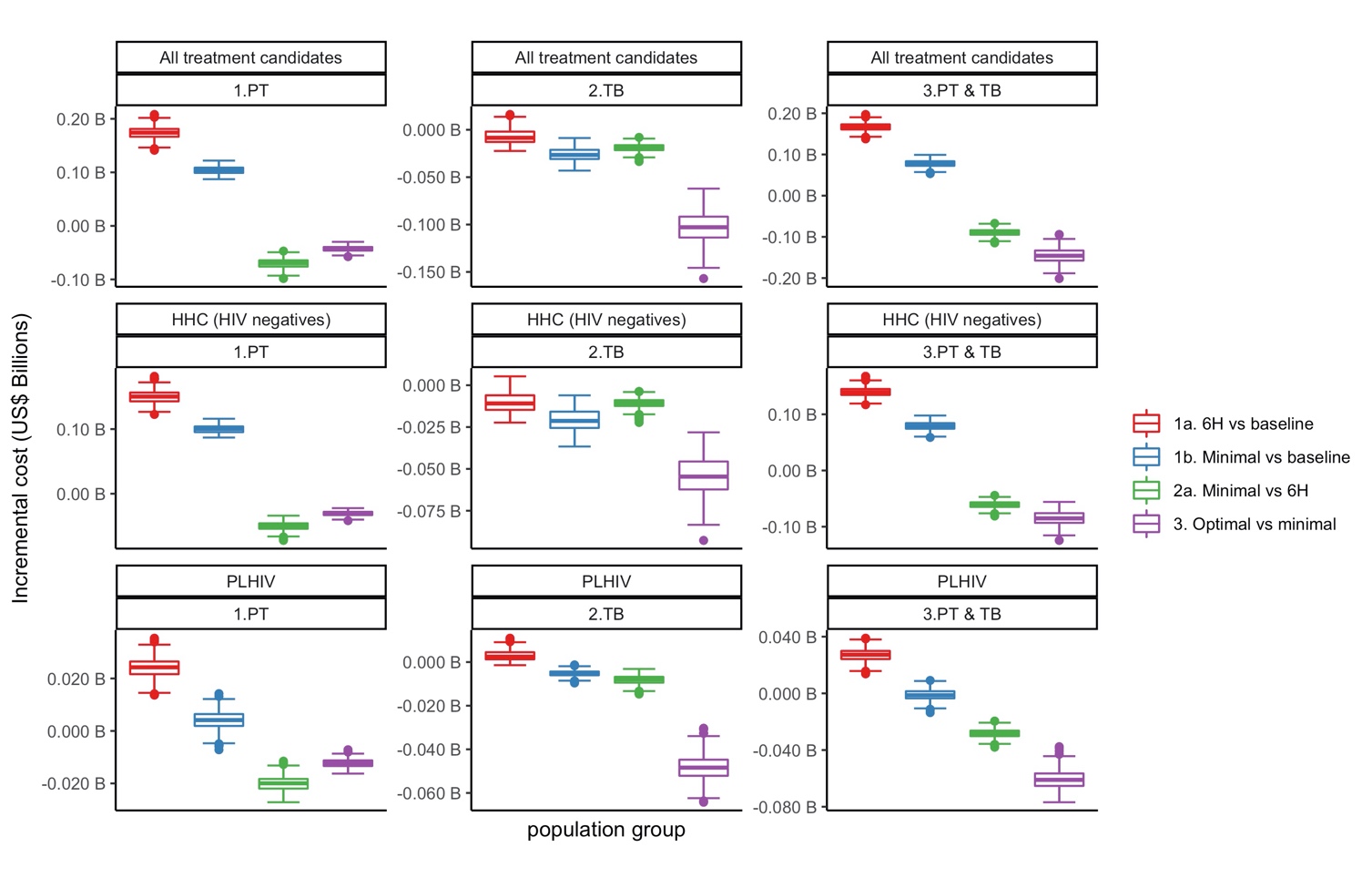
***

*Uncertainty ranges (UR), six-months of daily isoniazid (6H), Tuberculosis preventive treatment (PT), tuberculosis (TB), Household TB contacts (HHC), persons living with HIV (PLHIV)*

##### **Fig B.** Incremental costs discounted at a 4% annual rate, Brazil

***
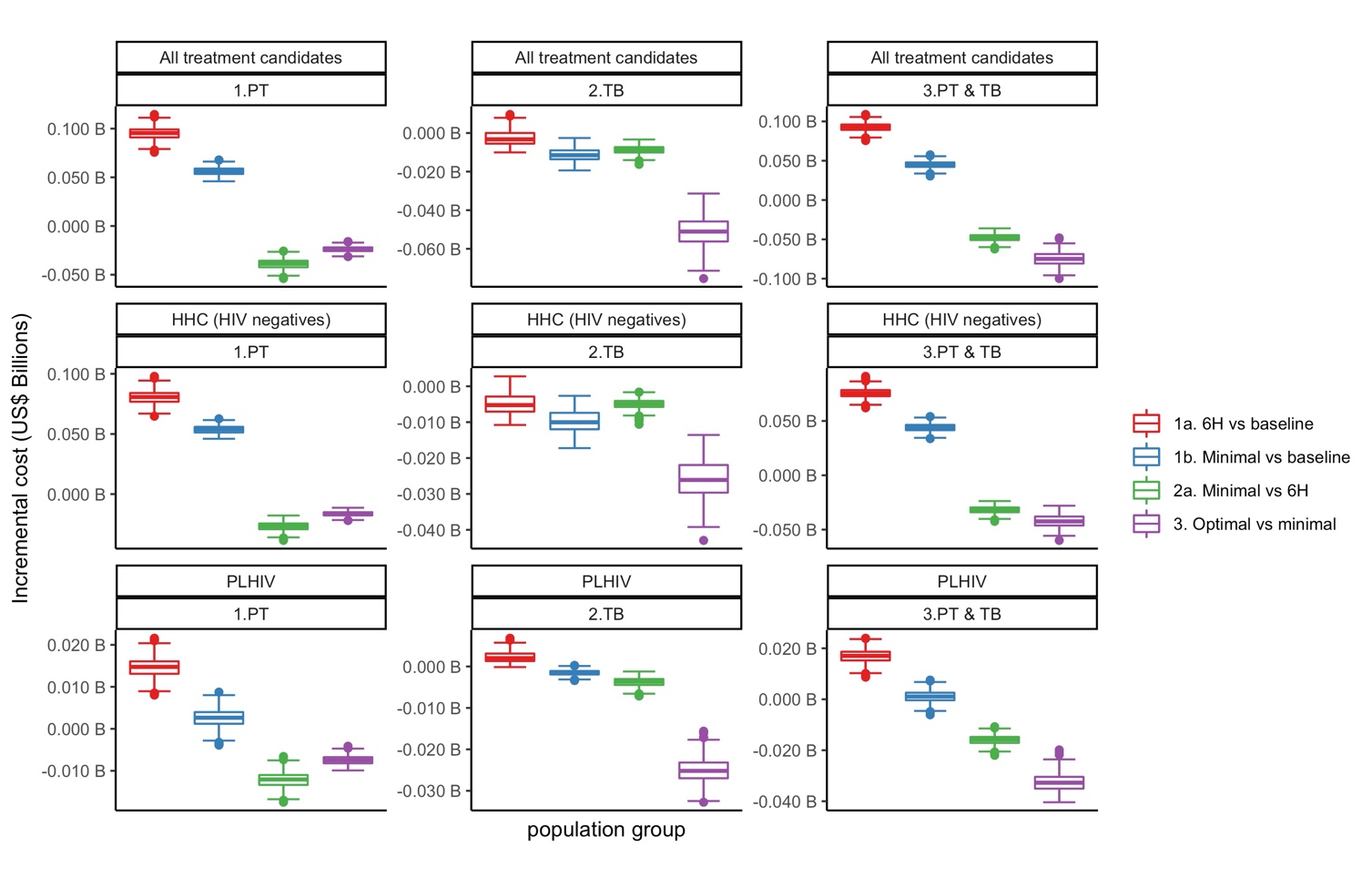
***

*Uncertainty ranges (UR), six-months of daily isoniazid (6H), Tuberculosis preventive treatment (PT), tuberculosis (TB), Household TB contacts (HHC), persons living with HIV (PLHIV)*

##### **Fig C.** Incremental undiscounted costs, South Africa

***
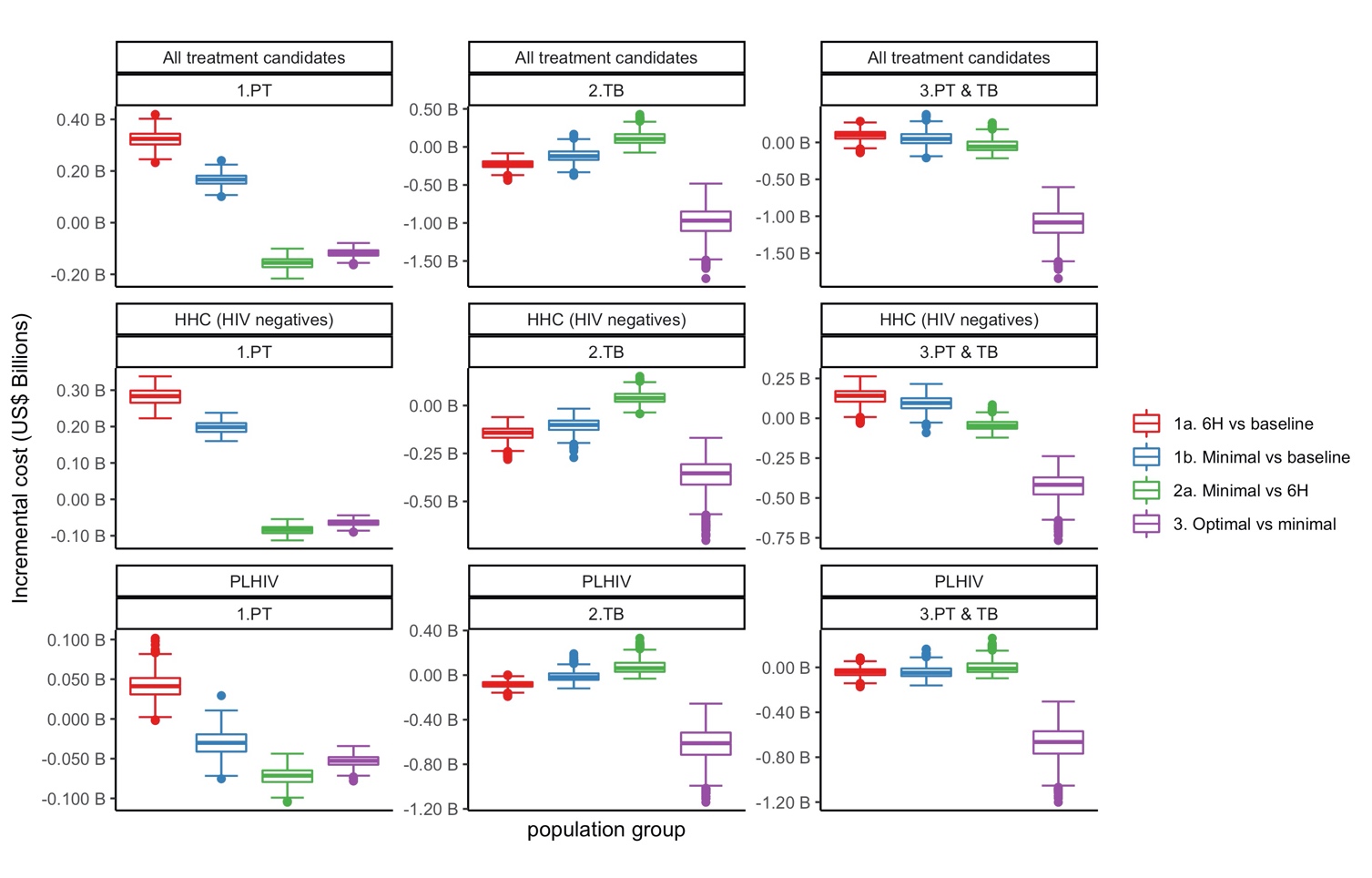
***

*Uncertainty ranges (UR), six-months of daily isoniazid (6H), Tuberculosis preventive treatment (PT), tuberculosis (TB), Household TB contacts (HHC), persons living with HIV (PLHIV)*

##### **Fig D.** Incremental costs discounted at a 4% annual rate, South Africa

***
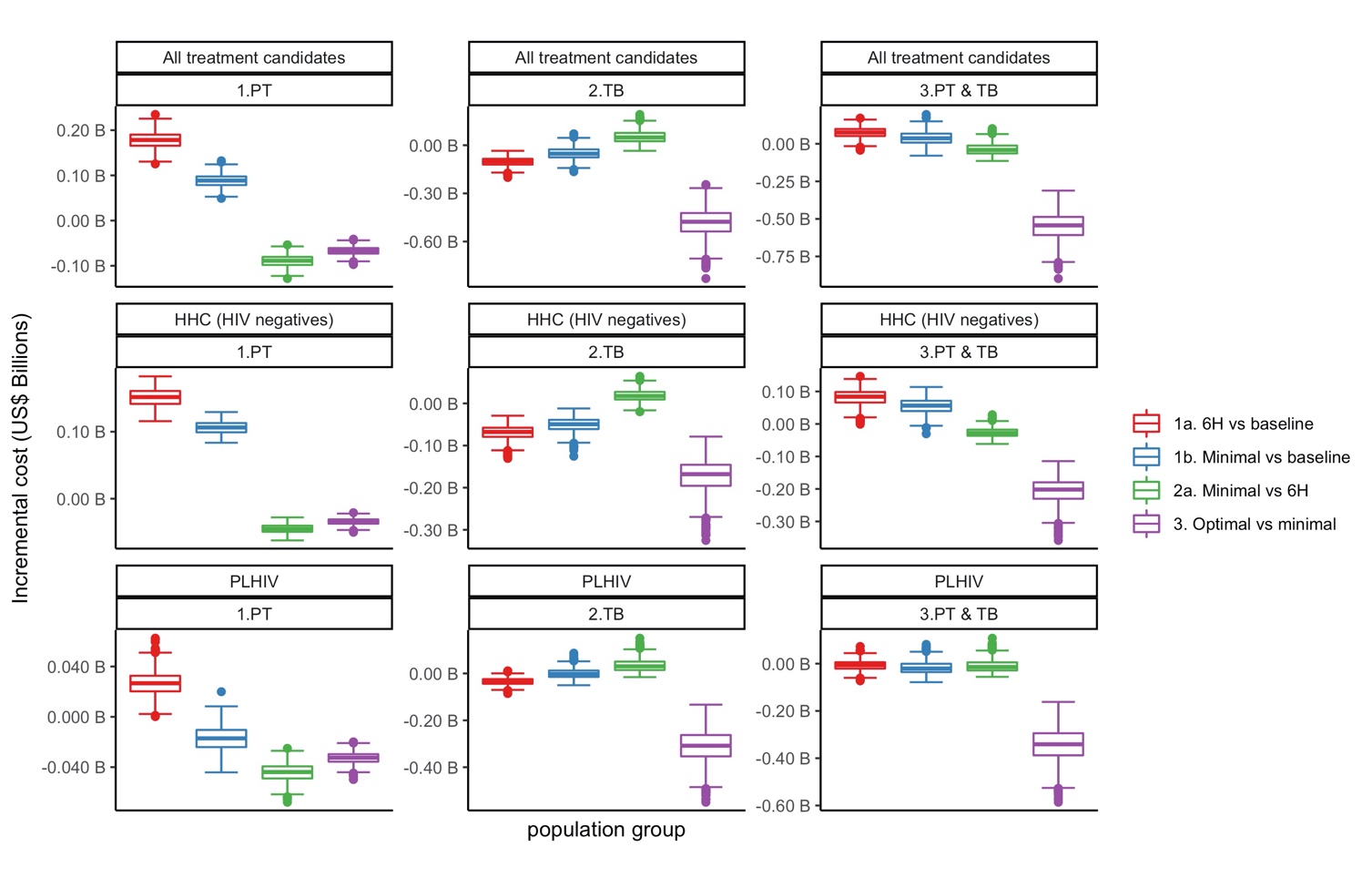
***

*Uncertainty ranges (UR), six-months of daily isoniazid (6H), Tuberculosis preventive treatment (PT), tuberculosis (TB), Household TB contacts (HHC), persons living with HIV (PLHIV)*

##### **Fig E.** Threshold analysis of TPT, Brazil, Break-even point stratified by treatment group^*^

**
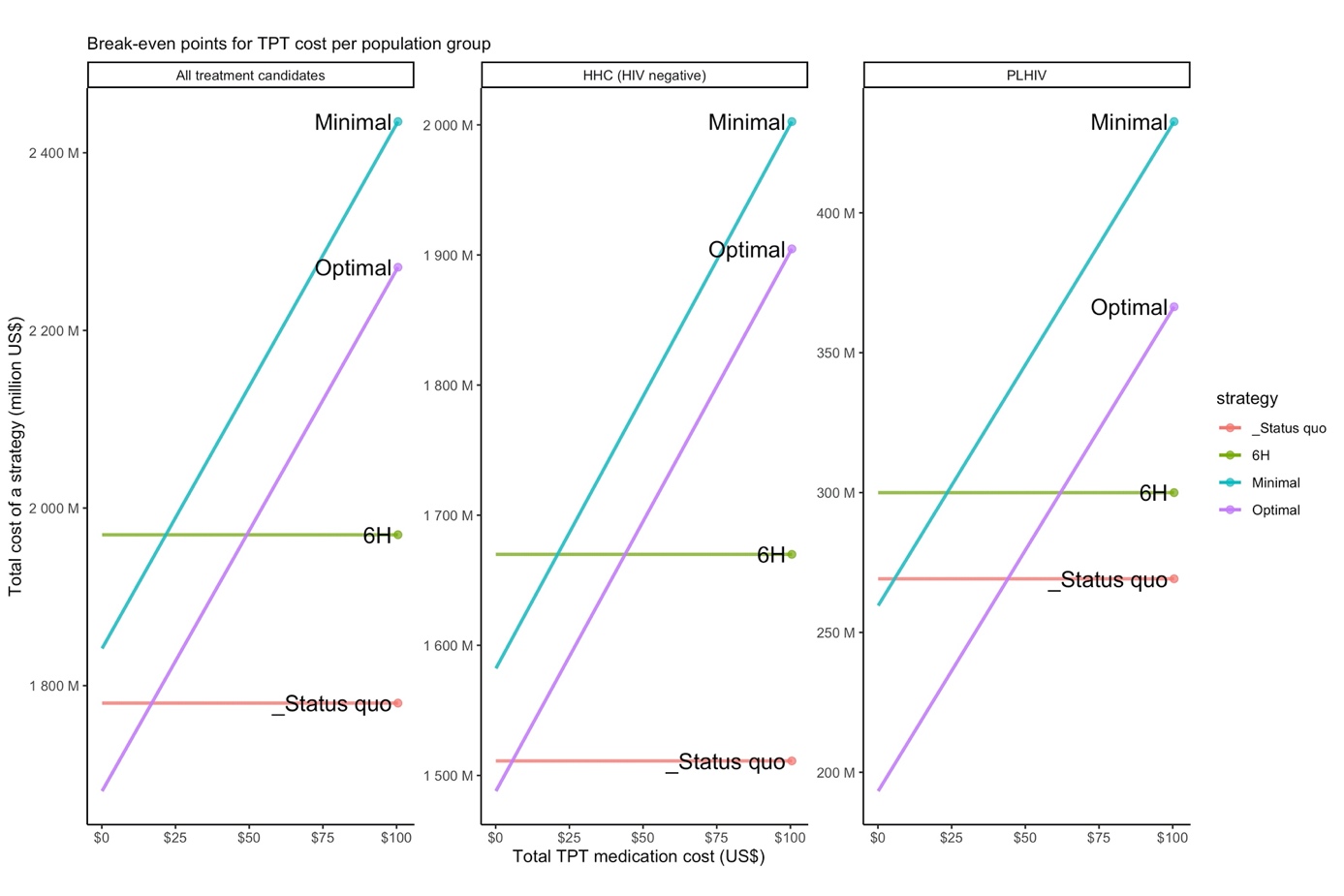
**

*^*^ The medication cost for 6H is kept constant as we vary the novel regimen cost to get the break-even point, TB management is done by a mixed model of SAT-DOT*

*Tuberculosis preventive treatment (TPT) , six-months of daily isoniazid (6H), results are discounted at a 3% annual rate*

##### **Fig F.** Threshold analysis of TPT, South Africa, Break-even point stratified by treatment group^*^

**
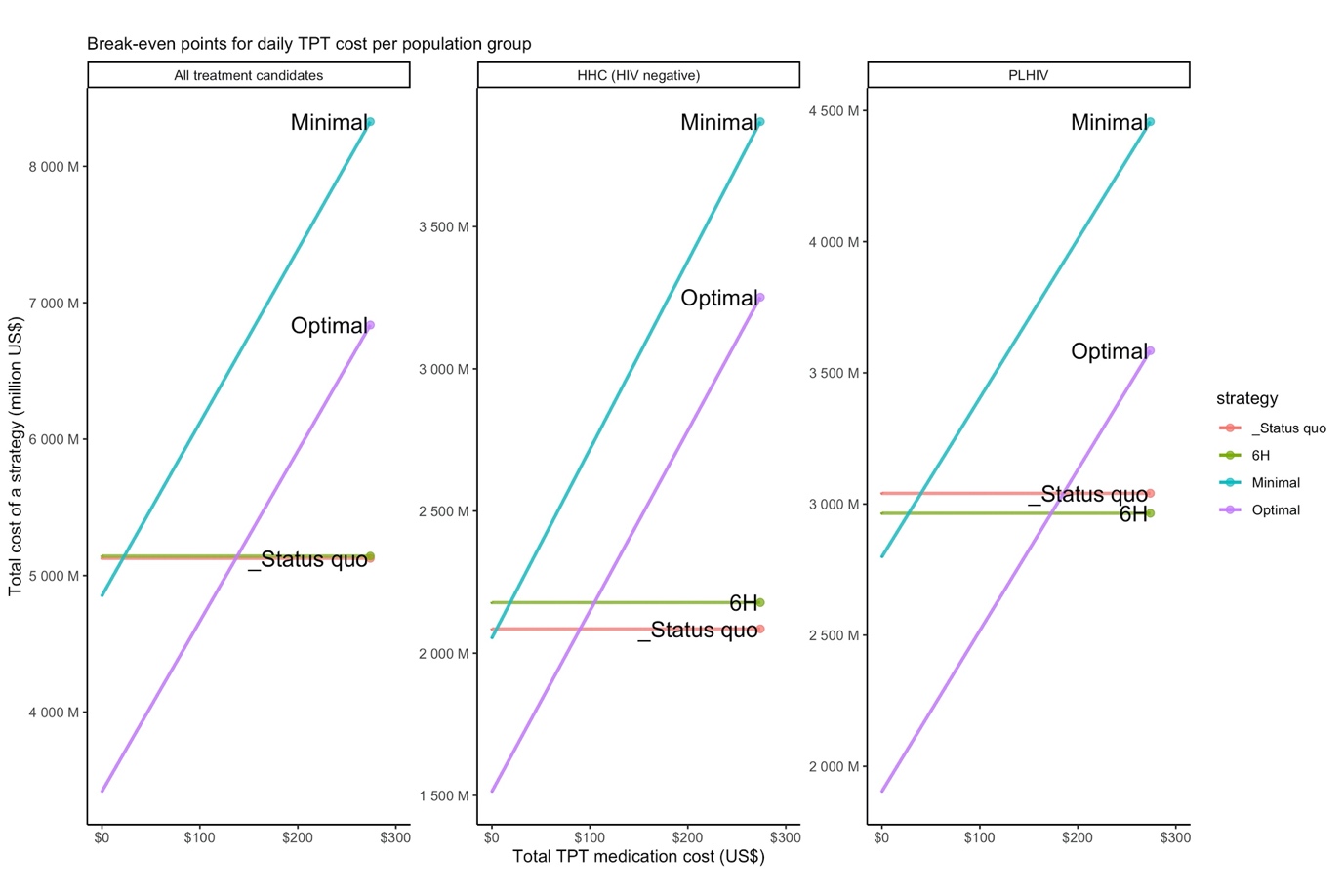
**

*^*^ The medication cost for 6H is kept constant as we vary the novel regimen cost to get the break-even point, TB management is done by a mixed model of SAT-DOT*

*Tuberculosis preventive treatment (TPT),, six-months of daily isoniazid (6H), results are discounted at a 3% annual rate*

##### **Fig G.** Varying the Rifampicin-resistance barrier, Brazil, (2020 – 2035) *


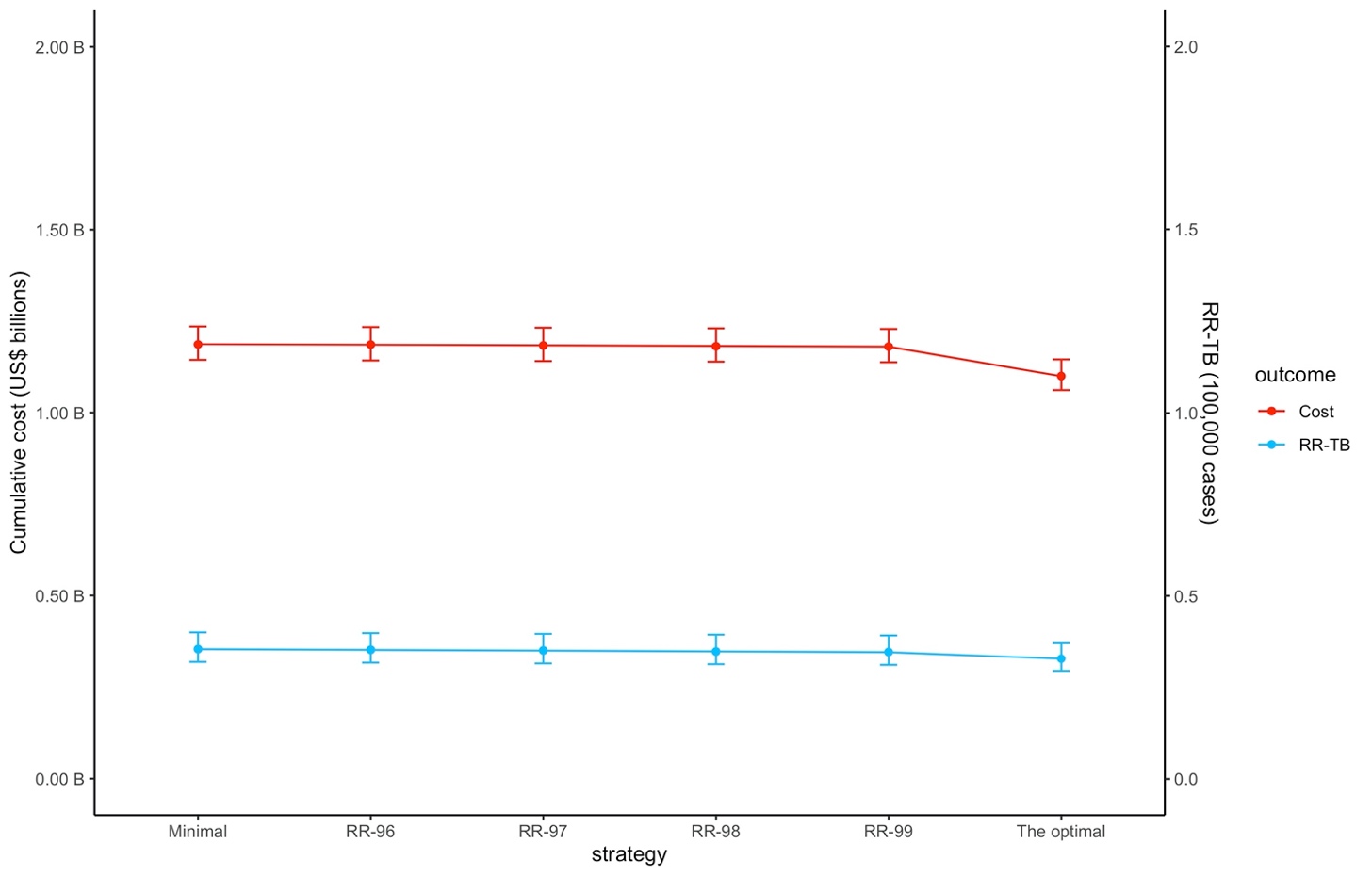


*^*^ e.g., RR-96 refers to 96% RR barrier (the minimal regimen has 95% RR barrier), Cost estimates for TB disease treatment by combined SAT-DOT, results are discounted at a 3% annual rate, rifampicin-resistant tuberculosis (RR-TB)*

##### **Fig H.** Rifampicin-resistance barrier, South Africa, (2020 – 2035) *


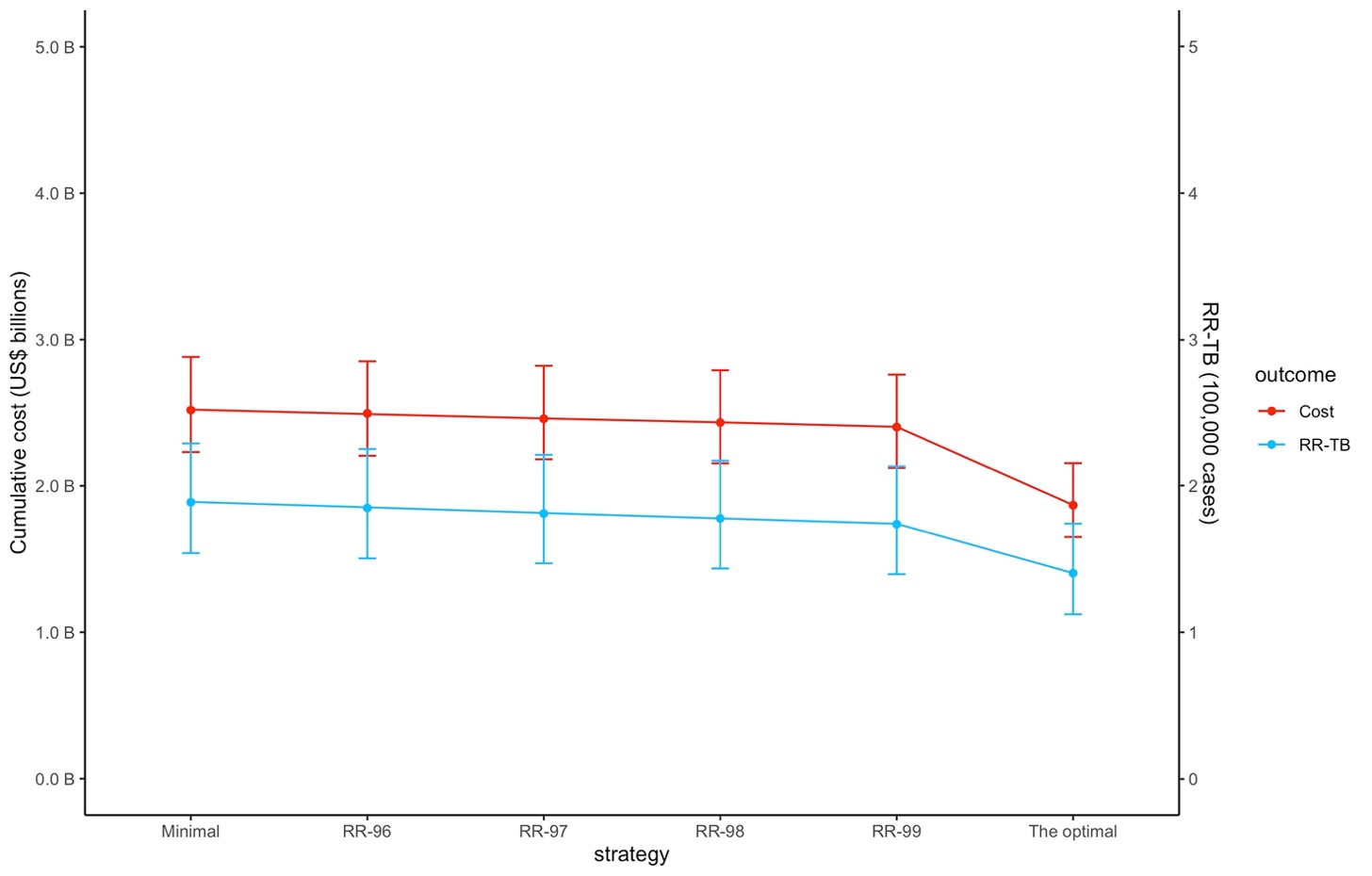


*^*^ e.g., RR-96 refers to 96% RR barrier (the minimal regimen has 95% RR barrier) , Cost estimates for TB disease treatment by combined SAT-DOT, results are discounted at a 3% annual rate, rifampicin-resistant tuberculosis (RR-TB)*

##### **Fig I.** Varying the effect of TPT regimens on RR strains, Brazil *


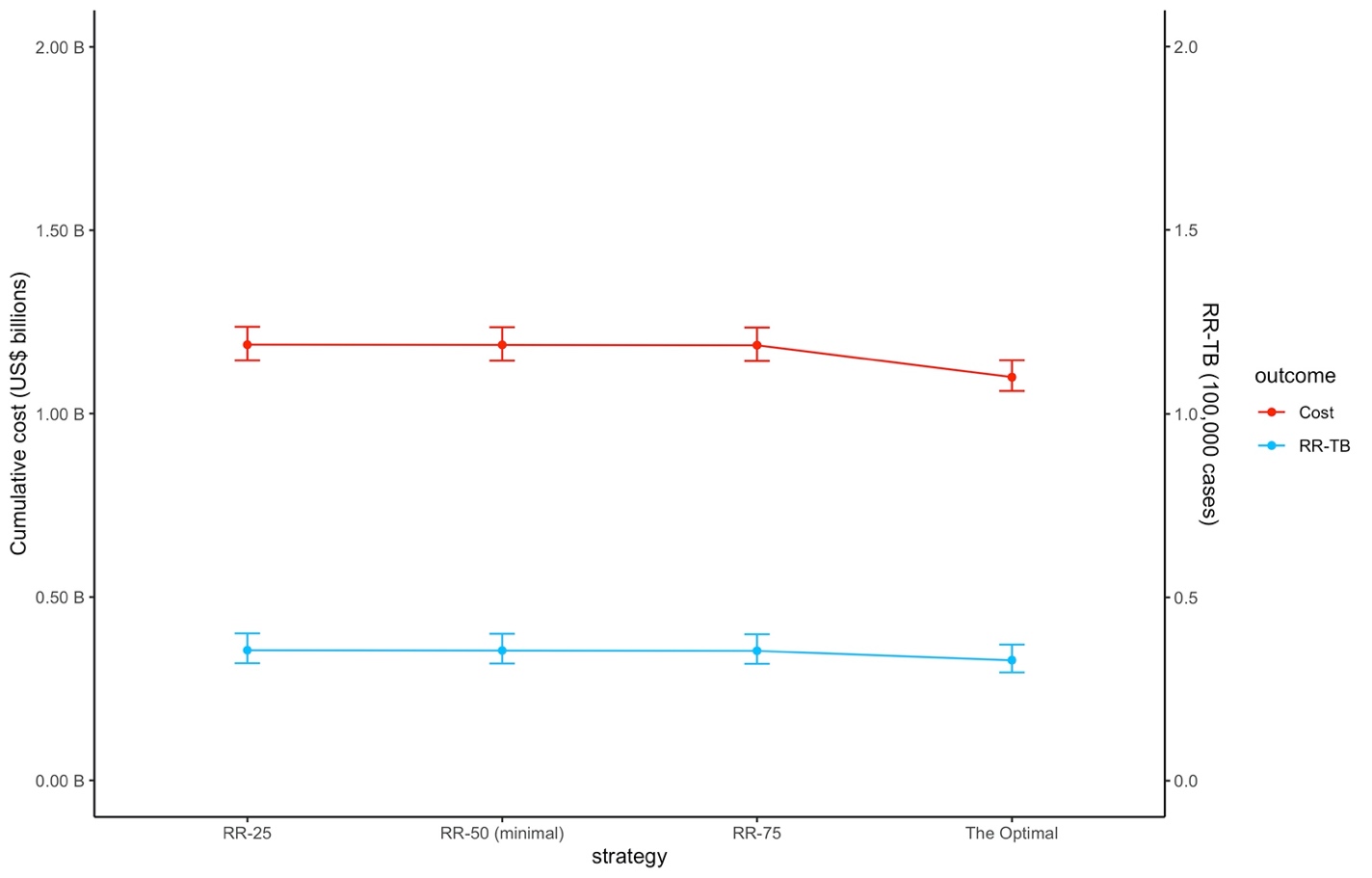


*^*^ e.g., RR-25 refers to 25% effect on RR strains (base case analysis considered a 50% effect on rifampicin resistant strains compared to their efficacy against drug susceptible strains), rifampicin-resistant tuberculosis (RR-TB), results are discounted at a 3% annual rate*

##### **Fig J.** The effect of TPT regimens on RR strains, South Africa *


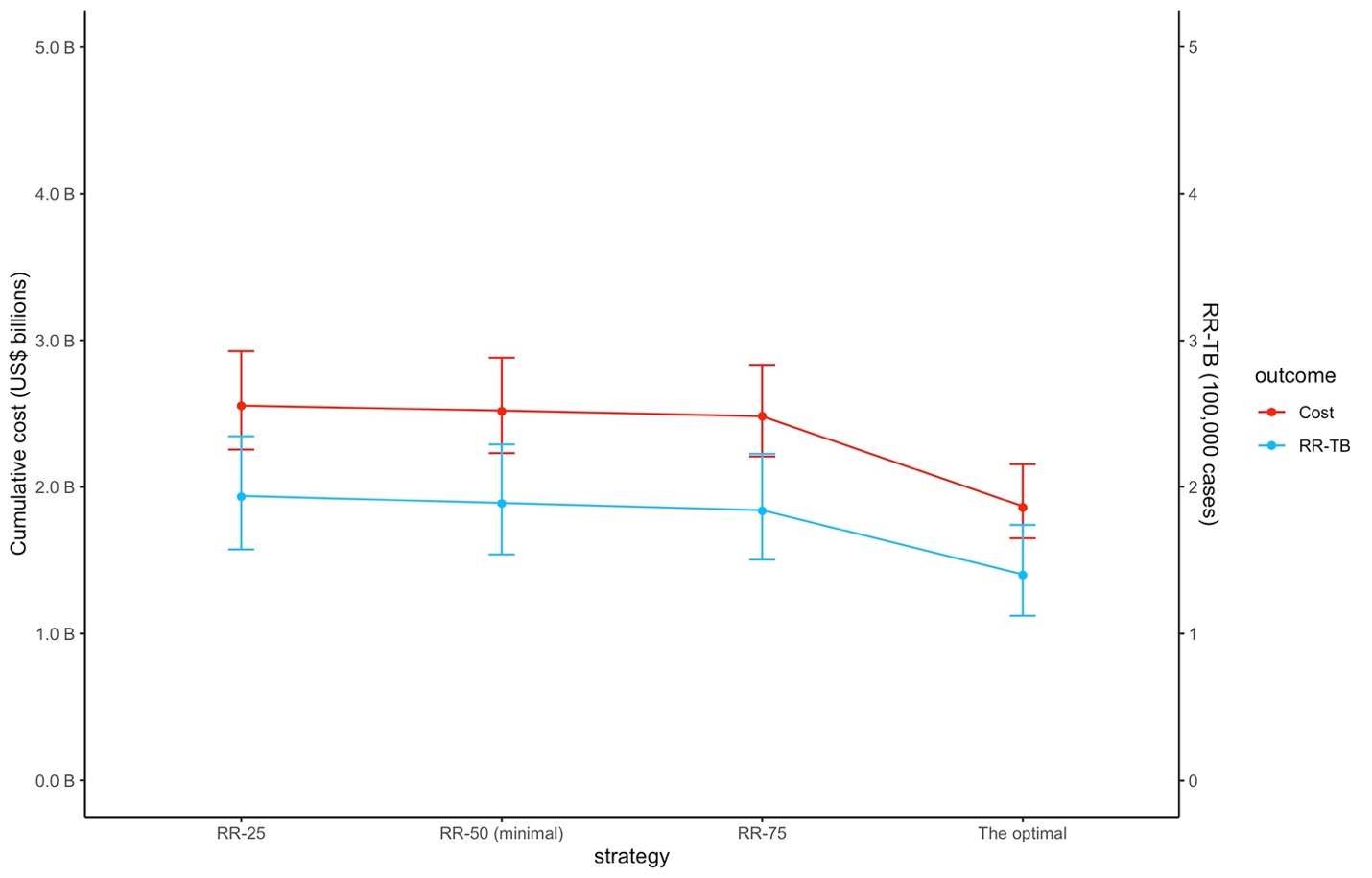


*^*^ e.g., RR-25 refers to 25% effect on RR strains (base case analysis considered a 50% effect on rifampicin resistant strains compared to their efficacy against drug susceptible strains), rifampicin-resistant tuberculosis (RR-TB), results are discounted at a 3% annual rate*

##### **Table L.** Cost estimates (95% UR) for TPT, and for TB disease treatment by universal DOT at 3% annual discount rate, Brazil and South Africa, 2020-2035 (US dollars in Millions)

| **Cost (95% UR) of TPT and TB management by SAT-DOT (in US dollars in Millions), 2020-2035** | | | | |
| --- | --- | --- | --- | --- |
| **Cost** | **Status quo** | **Scale-up 6H** | **Minimal - TPT** | **Optimal - TPT** |
| **Brazil** | | | | |
| TPT | **$25**  ($19, $30) | **$134**  ($118, $148) | **$90**  ($79, $99) | $62  ($55, $69) |
| Diagnosis and treatment of DS-TB | **$964**  ($868, $1 058) | **$961**  ($870, $1 052) | **$936**  ($846, $1 024) | $892  ($808, $973) |
| Diagnosis and treatment of RR-TB | **$370**  ($260, $518) | **$369**  ($259, $518) | **$379**  ($267, $529) | $352  ($249, $492) |
| Diagnosis and treatment of TB disease | **$1 334**  ($1 192, $1 492) | **$1 330**  ($1 193, $1 485) | **$1 315**  ($1 178, $1 473) | $1 244  ($1 122, $1 389) |
| Total cost (TPT & TB) | **$1 359**  ($1 215, $1 518) | **$1 464**  ($1 322, $1 621) | **$1 405**  ($1 266, $1 563) | $1 307  ($1 182, $1 451) |
| **South Africa** | | | | |
| TPT | **$145**  ($113, $172) | **$349**  ($290, $401) | **$248**  ($207, $285) | $172  ($144, $196) |
| Diagnosis and treatment of DS-TB | **$1 920**  ($1 560, $2 340) | **$1 792**  ($1 463, $2 159) | **$1 584**  ($1 280, $1 917) | $1 166  (951, $1 387) |
| Diagnosis and treatment of RR-TB | **$1 614**  ($866, $2 780) | **$1 542**  ($832, $2 653) | **$1 719**  ($945, $2 886) | $1 311  ($692, $2 279) |
| Diagnosis and treatment of TB disease | **$3 534**  ($2 692, $4 646) | **$3 335**  ($2 543, $4 398) | **$3 303**  ($2 452, $4 456) | $2 477  ($1 820, $3 415) |
| Total cost (TPT & TB) | $3 678  ($2 829, $4 791) | $3 684  ($2 888, $4 752) | $3 551  ($2 691, $4 703) | $2 649  ($1 996, $3 585) |

*Uncertainty ranges (UR), Tuberculosis preventive treatment (TPT), directly observed treatment (DOT), six-months of daily isoniazid (6H), tuberculosis (TB), persons living with HIV (PLHIV))*

##### **Table M**. Incremental costs (95% UR) for TPT, and for TB disease treatment by universal DOT at 3% annual discount rate, Brazil and South Africa, 2020-2035 (US dollars in Millions) *

|  | **6H vs Status quo ^**^** | **Minimal vs Status quo** | **Minimal vs 6H** | **Optimal vs 6H** | **Optimal vs minimal** |
| --- | --- | --- | --- | --- | --- |
| **Brazil** | | | | | |
| **All treatment candidates** | | | | | |
| Cost of TPT | **$109**  ($97, $122) | **$65**  ($57, $73) | **-$45**  (-$55, -$34) | **-$72**  (-$82, -$61) | **-$27**  (-$33, -$21) |
| Cost of diagnosis and treatment of TB disease | **-$4**  (-$12, $8) | **-$19**  (-$27, -$10) | **-$15**  (-$20, -$10) | **-$85**  (-$103, -$66) | **-$71**  (-$88, -$52) |
| Total cost (TPT & TB) | **$105**  ($94, $117) | **$46**  ($34, $56) | **-$59**  (-$70, -$50) | **-$157**  (-$180, -$133) | **-$98**  (-$117, -$78) |
| **Household contacts (HIV negative)** | | | | | |
| Cost of TPT | **$93**  ($83, $103) | **$62**  ($56, $69) | **-$31**  (-$38, -$24) | **-$50**  (-$57, -$43) | **-$19**  (-$23, -$15) |
| Cost of diagnosis and treatment of TB disease | **-$7**  (-$13, $1) | **-$15**  (-$23, -$7) | **-$8**  (-$12, -$5) | **-$44**  (-$59, -$27) | **-$36**  (-$48, -$21) |
| Total cost (TPT & TB) | **$86**  ($77, $95) | **$47**  ($37, $56) | **-$39**  (-$47, -$32) | **-$94**  (-$110, -$77) | **-$55**  (-$68, -$40) |
| **PLHIV** | | | | | |
| Cost of TPT | **$16**  ($11, $21) | **$3**  (-$2, $7) | **-$14**  (-$17, -$10) | **-$22**  (-$26, -$18) | **-$8**  (-$11, -$6) |
| Cost of diagnosis and treatment of TB disease | **$3**  ($0, $7) | **-$4**  (-$5, -$2) | **-$7**  (-$10, -$4) | **-$41**  (-$47, -$34) | **-$35**  (-$42, -$27) |
| Total cost (TPT & TB) | **$19**  ($13, $25) | **-$1**  (-$6, $4) | **-$20**  (-$25, -$16) | **-$63**  (-$72, -$53) | **-$43**  (-$51, -$34) |
| **South Africa** | | | | | |
| **All treatment candidates** | | | | | |
| Cost of TPT | **$204**  ($168, $243) | **$103**  ($76, $132) | **-$102**  (-$129, -$77) | **-$178**  (-$213, -$143) | **-$76**  (-$95, -$57) |
| Cost of diagnosis and treatment of TB disease | **-$199**  (-$301, -$118) | **-$230**  (-$360, -$102) | **-$32**  (-$127, $84) | **-$858**  (-$1 080, -$655) | **-$826**  (-$1 118, -$596) |
| Total cost (TPT & TB) | **$6**  (-$107, $104) | **-$128**  (-$268, $9) | **-$133**  (-$225, -$17) | **-$1 035**  (-$1 258, -$831) | **-$902**  (-$1 192, -$668) |
| **Household contacts (HIV negative)** | | | | | |
| Cost of TPT | **$175**  ($147, $200) | **$122**  ($103, $140) | **-$52**  (-$65, -$40) | **-$92**  (-$110, -$74) | **-$40**  (-$50, -$30) |
| Cost of diagnosis and treatment of TB disease | **-$131**  (-$195, -$79) | **-$137**  (-$218, -$69) | **-$5**  (-$49, $35) | **-$289**  (-$416, -$180) | **-$284**  (-$410, -$184) |
| Total cost (TPT & TB) | **$43**  (-$33, $107) | **-$14**  (-$103, $64) | **-$58**  (-$99, -$15) | **-$381**  (-$507, -$278) | **-$324**  (-$446, -$225) |
| **PLHIV** | | | | | |
| Cost of TPT | **$30**  ($11, $52) | **-$19**  (-$41, $2) | **-$49**  (-$64, -$36) | **-$86**  (-$105, -$67) | **-$36**  (-$46, -$27) |
| Cost of diagnosis and treatment of TB disease | **-$67**  (-$120, -$15) | **-$94**  (-$154, -$31) | **-$26**  (-$79, $56) | **-$568**  (-$739, -$407) | **-$542**  (-$780, -$347) |
| Total cost (TPT & TB) | **-$38**  (-$101, $25) | **-$113**  (-$180, -$40) | **-$76**  (-$129, $7) | **-$654**  (-$824, -$486) | **-$578**  (-$816, -$383) |

** TB management is done by universal DOT ** negative values indicate cost savings*

*Uncertainty ranges (UR), six-months of daily isoniazid (6H), Tuberculosis preventive treatment (TPT) ), six-months of daily isoniazid (6H), tuberculosis (TB), persons living with HIV (PLHIV)*

##### **Fig K.** Incremental costs, 10-years scale up of TPT, South Africa

***
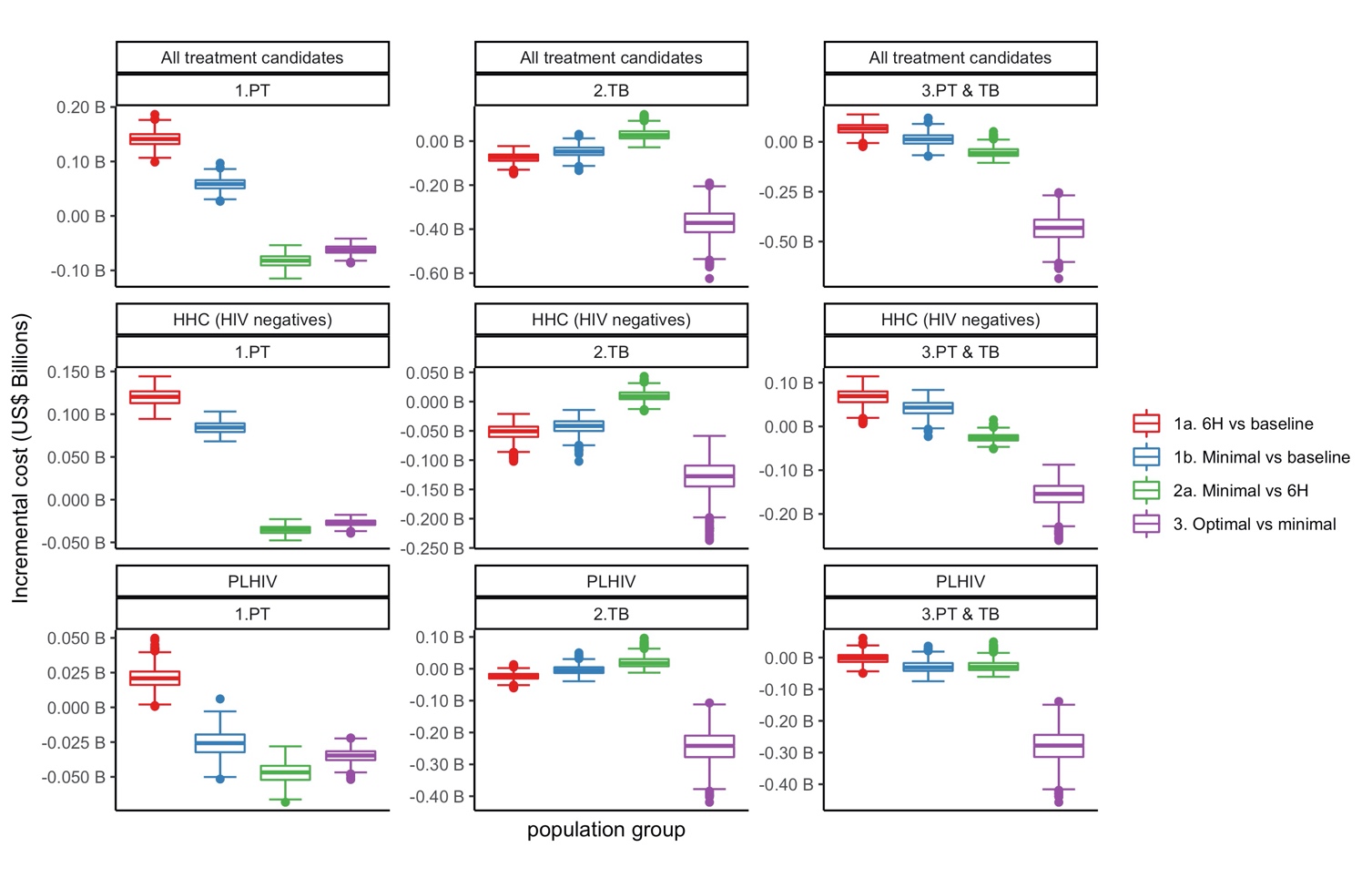
***

*Uncertainty ranges (UR), six-months of daily isoniazid (6H), Tuberculosis preventive treatment (PT), tuberculosis (TB), Household TB contacts (HHC), persons living with HIV (PLHIV)*

##### **Fig L.** Incremental costs, 16-years scale up of TPT, South Africa

***
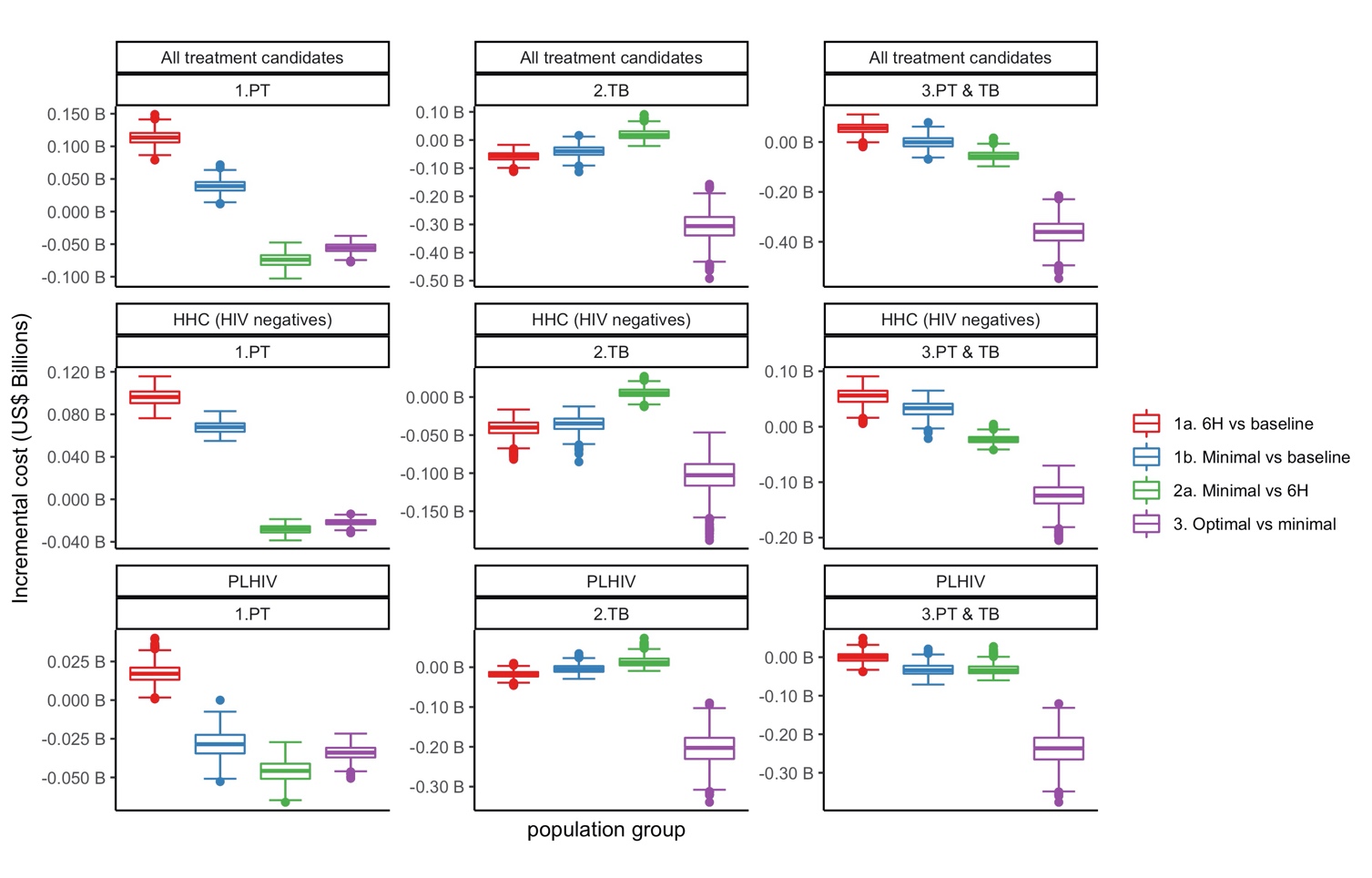
***

*Uncertainty ranges (UR), six-months of daily isoniazid (6H), Tuberculosis preventive treatment (PT), tuberculosis (TB), Household TB contacts (HHC), persons living with HIV (PLHIV)*

##### **Fig M.** Incremental cost-effectiveness planes, 10-years scale up of TPT, South Africa ^*^


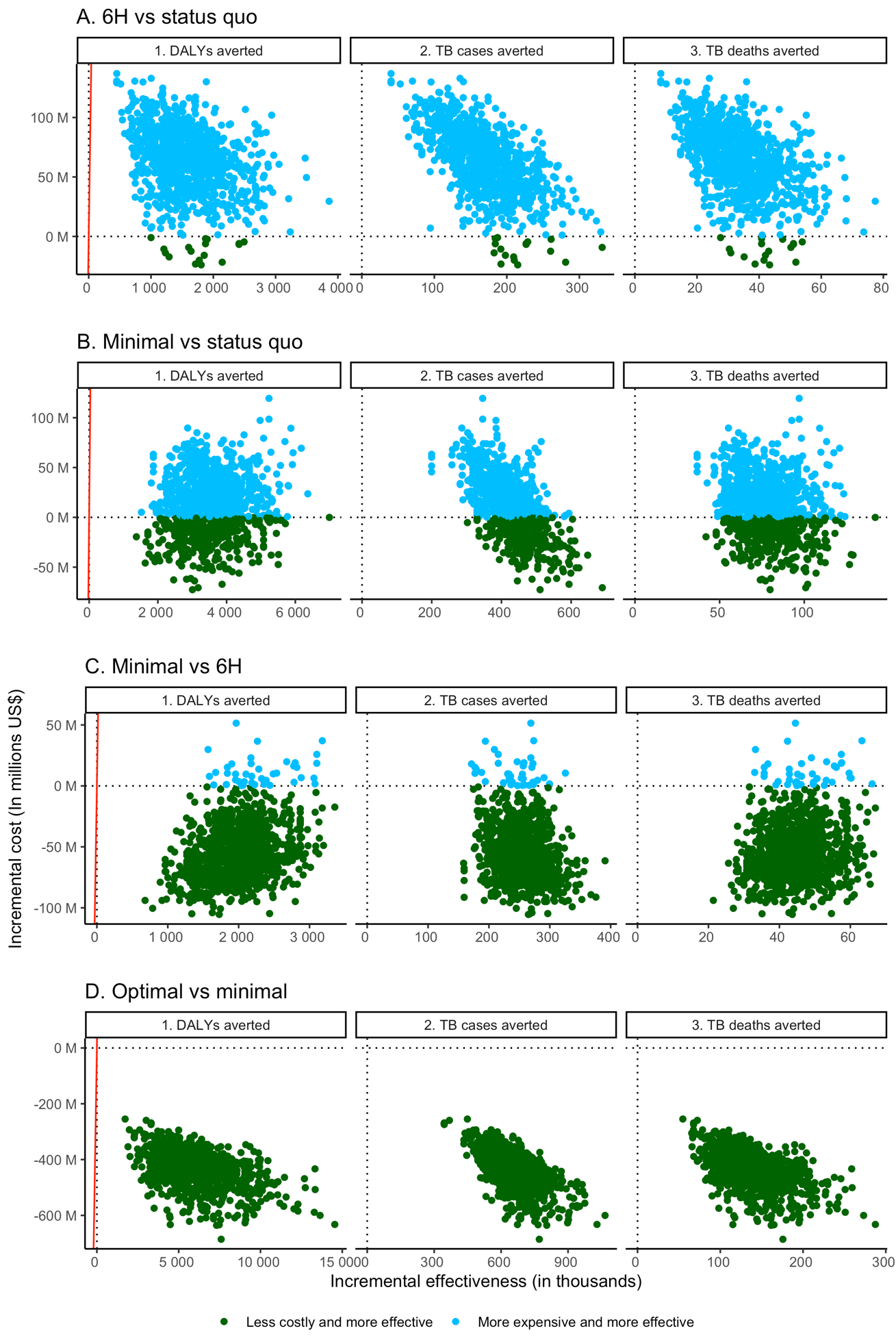


** Results are discounted at a 3% annual rate*

*(A) 6 months daily isoniazid (6H) vs. status quo. (B) Minimal regimen vs. status quo. (C) Minimal regimen vs. 6H. (D) Optimal vs. minimal regimen.*

*On the x-axis, negative values indicate poorer health outcomes. On the y-axis, negative values indicate cost savings. The red lines correspond to the willingness-to-pay threshold per DALY averted ($8 786/DALY averted). 6H = six months of daily isoniazid.*

##### **Fig N.** Incremental cost-effectiveness planes, 16-years scale up of TPT, South Africa ^*^


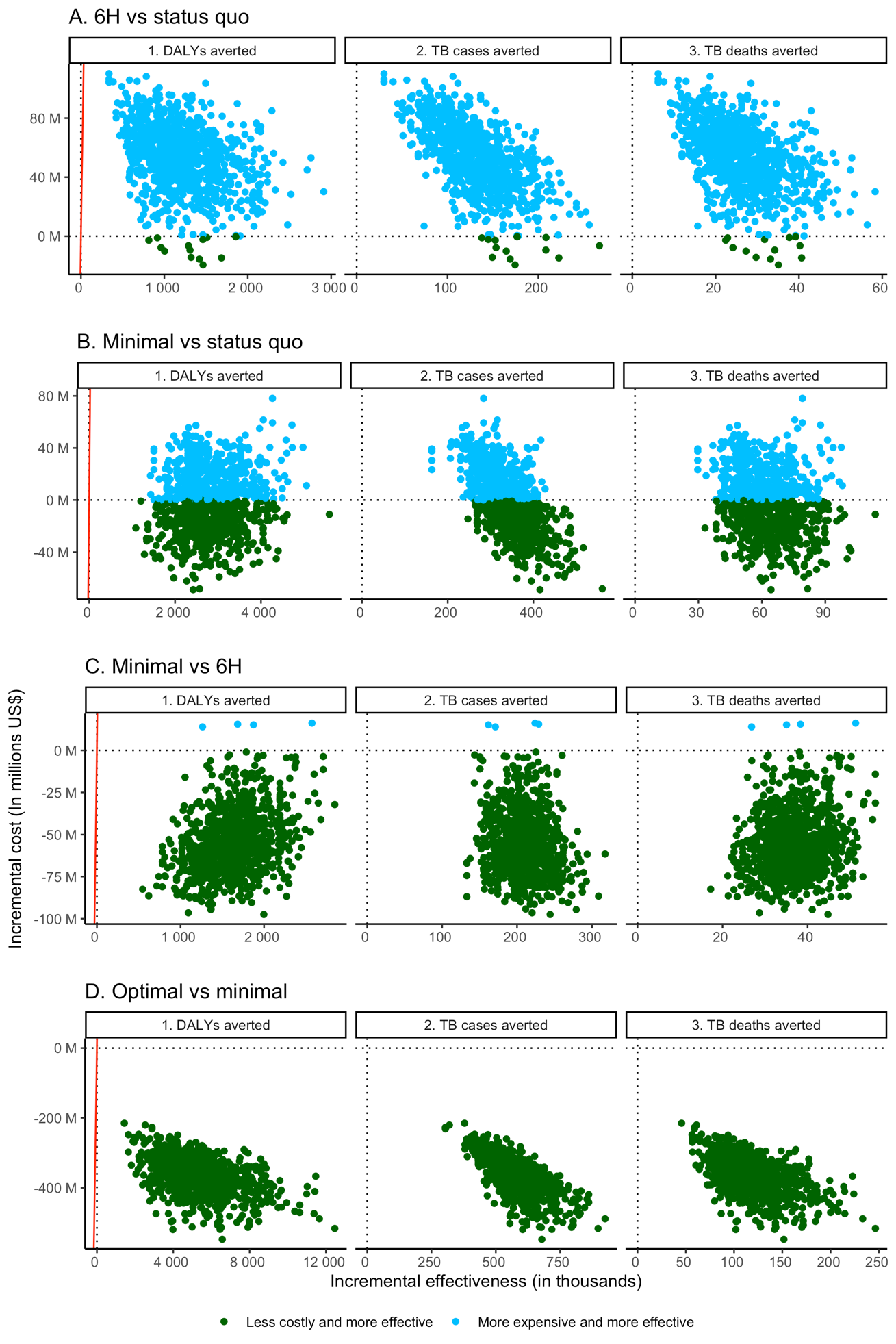


** Results are discounted at a 3% annual rate*

*(A) 6 months daily isoniazid (6H) vs. status quo. (B) Minimal regimen vs. status quo. (C) Minimal regimen vs. 6H. (D) Optimal vs. minimal regimen.*

*On the x-axis, negative values indicate poorer health outcomes. On the y-axis, negative values indicate cost savings. The red lines correspond to the willingness-to-pay threshold per DALY averted ($8 786/DALY averted). 6H = six months of daily isoniazid.*

##### **Table N.** Cumulative effectiveness projections with a 10-years scale up of TPT (in thousands), Brazil and South Africa, 2020–2035 ^*^

|  | Status quo | Scale up 6H | Minimal TPT | Optimal TPT |
| --- | --- | --- | --- | --- |
| Brazil | | | | |
| 1. No. of people initiating TPT | **669**  (507, 810) | **2 799**  (2 469, 3 021) | **2 795**  (2 466, 3 016) | 2 784  (2 462, 3 003) |
| 2. DS-TB cases | **1 093**  (1 017, 1 165) | **1 091**  (1 019, 1 161) | **1 075**  (1 002, 1 143) | 1 035  (971, 1 100) |
| 3. RR-TB cases | **35**  (25, 49) | **35**  (25, 49) | **36**  (26, 50) | 34  (24, 48) |
| 4. All TB cases (DS-TB & RR-TB) | **1 128**  (1 052, 1 199) | **1 126**  (1 054, 1 193) | **1 111**  (1 040, 1 177) | 1 069  (1 006, 1 131) |
| 5. Deaths | **123**  (115, 132) | **123**  (115, 131) | **122**  (114, 130) | 117  (110, 124) |
| 6. TB DALYs | **2 542**  (2 101, 3 118) | **2 535**  (2 093, 3 116) | **2 512**  (2 074, 3 084) | 2 393  (1 979, 2 952) |
| South Africa | | | | |
| 1. No. of people initiating TPT | **4 109**  (3 252, 4 852) | **8 493**  (7 180, 9 544) | **8 454**  (7 155, 9 475) | 8 358  (7 072, 9 314) |
| 2. DS-TB cases | **3 479**  (2 805, 4 243) | **3 317**  (2 709, 3 998) | **3 051**  (2 460, 3 694) | 2 427  (1 992, 2 892) |
| 3. RR-TB cases | **184**  (98, 316) | **178**  (95, 305) | **191**  (104, 320) | 160  (85, 277) |
| 4. All TB cases (DS-TB & RR-TB) | **3 663**  (3 002, 4 393) | **3 495**  (2 873, 4 149) | **3 242**  (2 654, 3 859) | 2 588  (2 153, 3 024) |
| 5. Deaths | **843**  (548, 1 201) | **809**  (530, 1 153) | **764**  (496, 1 095) | 625  (417, 889) |
| 6. TB DALYs | 36 682  (19 459, 58 024) | 35 119(18 463, 55 562) | 33 130  (17 137, 53 187) | 26 952  (14 163, 42 652) |

** The discount factor applied was 1/(1+D)^t^, where D is the 3% discount rate that is applied to each period t=year, TB disease management by a mixed model of SAT-DOT*

*Uncertainty ranges (UR), Tuberculosis preventive treatment (TPT), six-months of daily isoniazid (6H), drug-susceptible tuberculosis (DS-TB), rifampicin-resistant tuberculosis (RR-TB), disability-adjusted life years (TB DALYs), persons living with HIV (PLHIV)*

##### **Table O.** Cumulative effectiveness projections with a 16-years’ time horizon scale up of TPT (in thousands), Brazil and South Africa, 2020–2035 ^*^

|  | Status quo | Scale up 6H | Minimal TPT | Optimal TPT |
| --- | --- | --- | --- | --- |
| Brazil | | | | |
| No. of people initiating TPT | **197**  (144, 257) | **3 565**  (3 251, 3 854) | **3 551**  (3 251, 3 832) | 3 522  (3 220, 3 790) |
| 2. DS-TB cases | **-5**  (-13, 7) | **-35**  (-44, -25) | **-30**  (-36, -25) | -83  (-100, -66) |
| 3. RR-TB cases | **-0.07**  (-0.27, 0.19) | **0.90**  (0.71, 1.14) | **0.96**  (0.74, 1.27) | -2  (-3, -1) |
| TB cases (DS-TB & RR-TB) | **1 127**  (1 052, 1 195) | **1 119**  (1 053, 1 184) | **1 084**  (1 022, 1 146) | 1 021  (962, 1 085) |
| Deaths | **125**  (116, 133) | **124**  (116, 131) | **120**  (113, 127) | 113  (106, 119) |
| TB DALYs | **2 626**  (2 138, 3 167) | **2 601**  (2 114, 3 145) | **2 526**  (2 062, 3 051) | 2 338  (1 913, 2 818) |
| South Africa | | | | |
| No. of people initiating TPT | **4 151**  (3 417, 4 856) | **10 757**  (9 103, 11 844) | **10 717**  (9 081, 11 760) | 10 586  (9 034, 11 608) |
| 2. DS-TB cases | **-5**  (-13, 7) | **-35**  (-44, -25) | **-30**  (-36, -25) | -83  (-100, -66) |
| 3. RR-TB cases | **-0.07**  (-0.27, 0.19) | **0.90**  (0.71, 1.14) | **0.96**  (0.74, 1.27) | -2  (-3, -1) |
| TB cases (DS-TB & RR-TB) | **3 614**  (2 984, 4 377) | **3 387**  (2 835, 4 033) | **3 028**  (2 535, 3 620) | 2 105  (1 801, 2 443) |
| Deaths | **827**  (574, 1 196) | **775**  (538, 1 133) | **707**  (485, 1 053) | 509  (366, 726) |
| TB DALYs | 36 018  (21 602, 58 088) | 33 650  (20 329, 54 618) | 30 236  (18 270, 50 034) | 21 440  (13 088, 34 583) |

** The discount factor applied was 1/(1+D)^t^, where D is the 3% discount rate that is applied to each period t=year, TB disease management by a mixed model of SAT-DOT*

*Uncertainty ranges (UR), Tuberculosis preventive treatment (TPT), six-months of daily isoniazid (6H), drug-susceptible tuberculosis (DS-TB), rifampicin-resistant tuberculosis (RR-TB), disability-adjusted life years (DALYs), persons living with HIV (PLHIV)*

##### **Fig O.** Incremental costs, 10-years scale up of TPT, Brazil

***
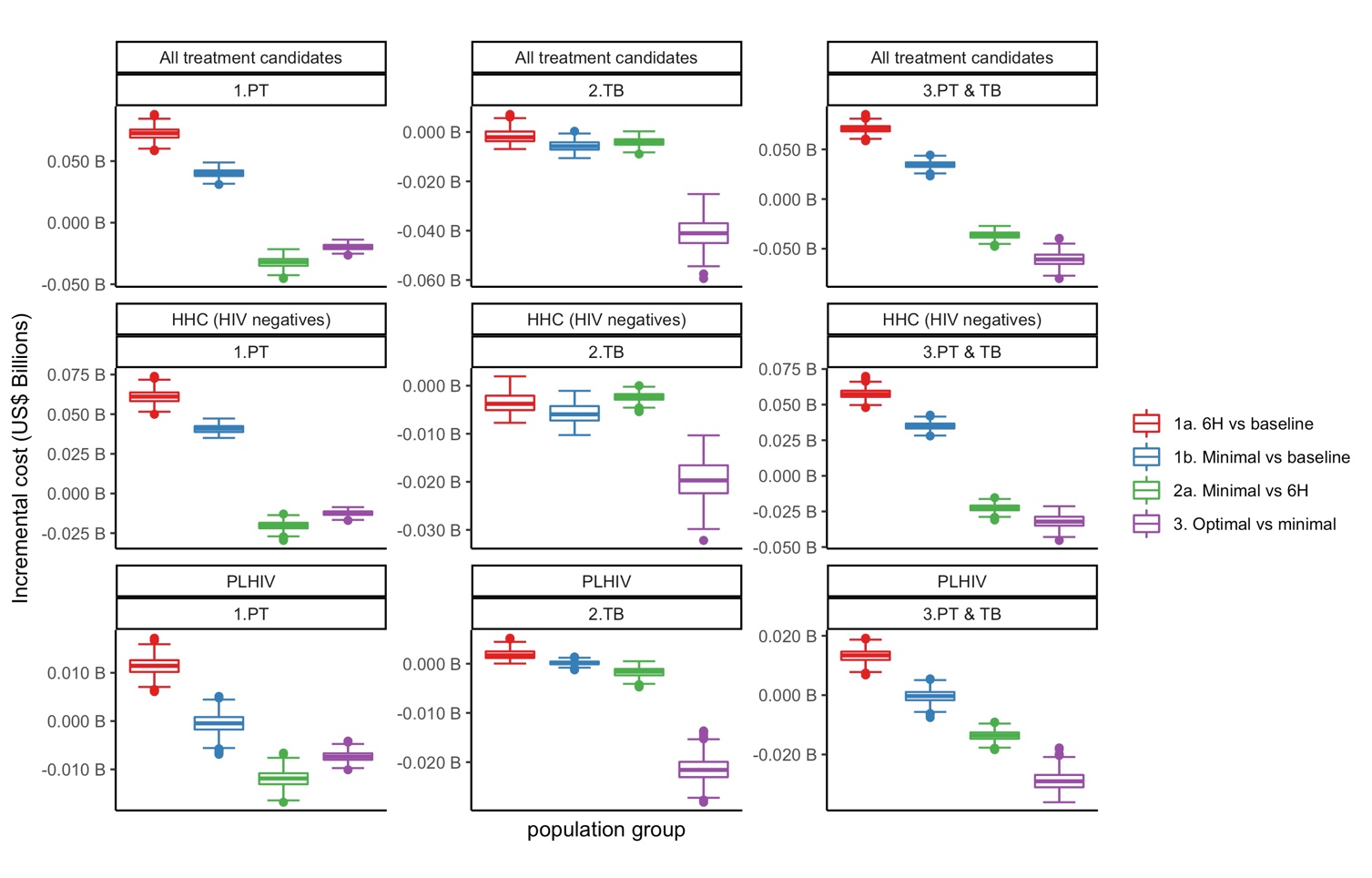
***

*Uncertainty ranges (UR), six-months of daily isoniazid (6H), Tuberculosis preventive treatment (PT), tuberculosis (TB), Household TB contacts (HHC), persons living with HIV (PLHIV)*

##### **Fig P.** Incremental costs, 16-years scale up of TPT, Brazil

***
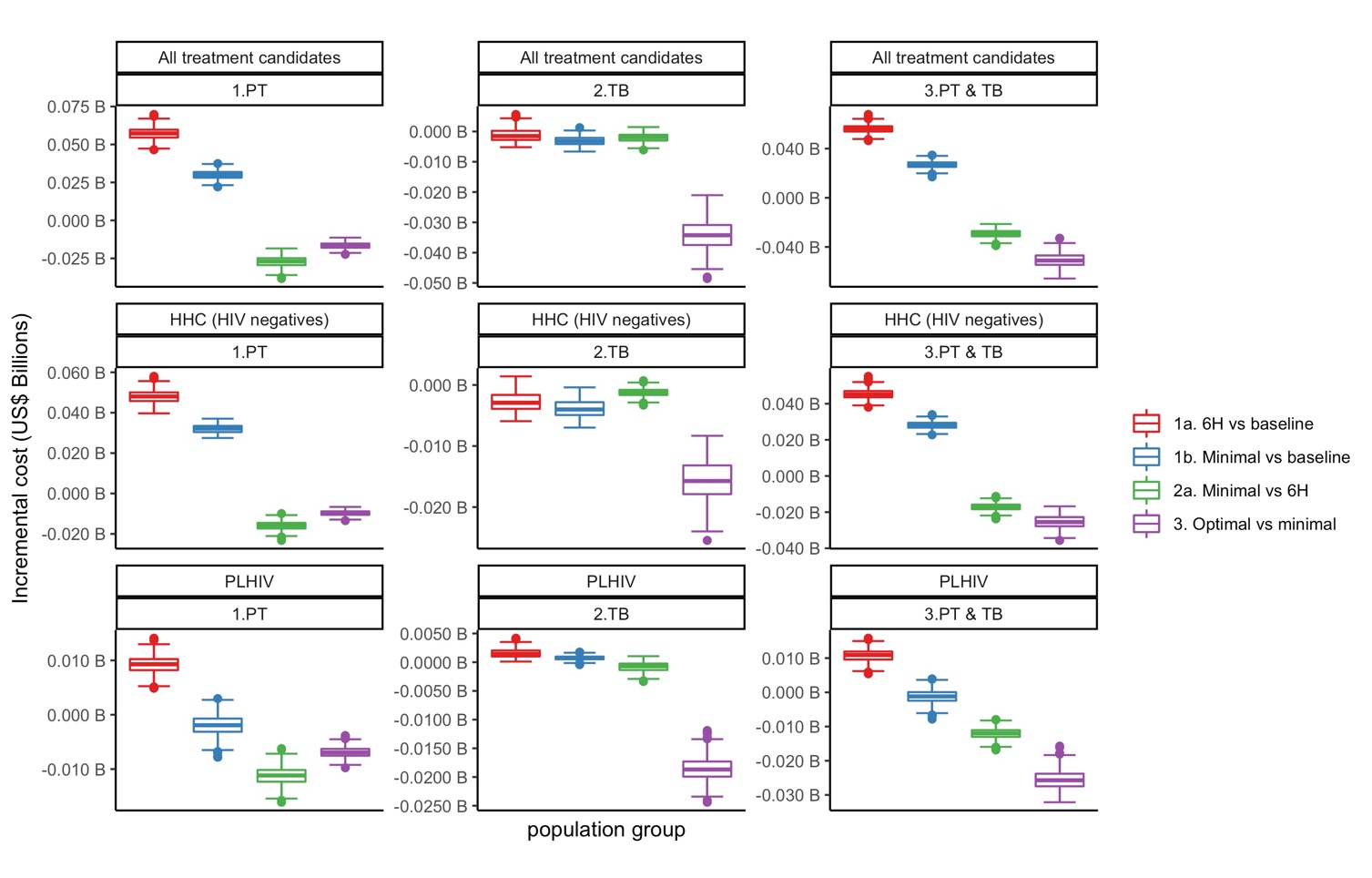
***

*Uncertainty ranges (UR), six-months of daily isoniazid (6H), Tuberculosis preventive treatment (PT), tuberculosis (TB), Household TB contacts (HHC), persons living with HIV (PLHIV)*

##### **Fig Q.** Incremental cost-effectiveness planes, 10-years scale up of TPT, Brazil ^*^


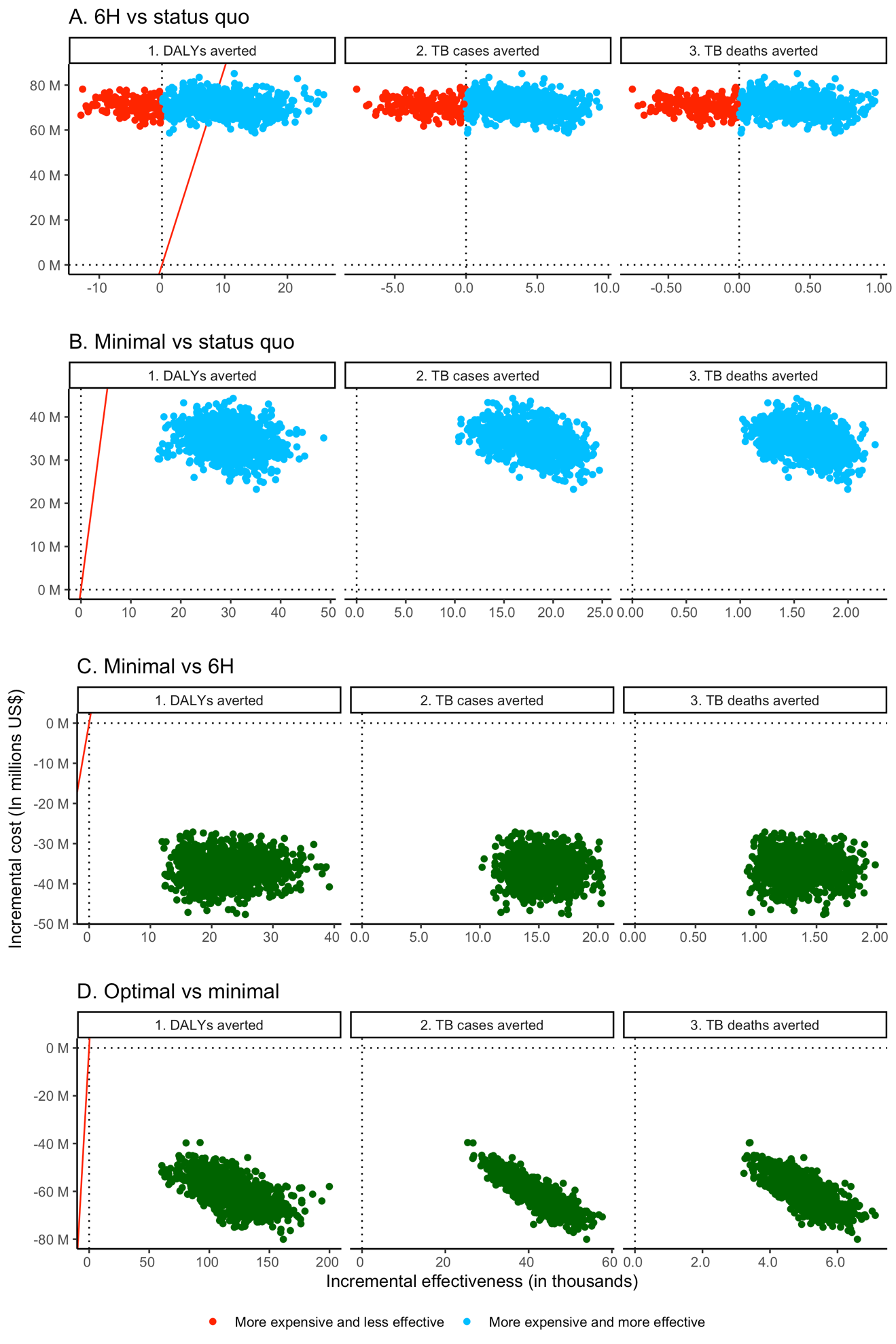


** Results are discounted at a 3% annual rate*

*(A) 6 months daily isoniazid (6H) vs. status quo. (B) Minimal regimen vs. status quo. (C) Minimal regimen vs. 6H. (D) Optimal vs. minimal regimen.*

*On the x-axis, negative values indicate poorer health outcomes. On the y-axis, negative values indicate cost savings. The red lines correspond to the willingness-to-pay threshold per DALY averted ($8 786/DALY averted). 6H = six months of daily isoniazid.*

##### **Fig R.** Incremental cost-effectiveness planes, 16-years scale up of TPT, Brazil ^*^


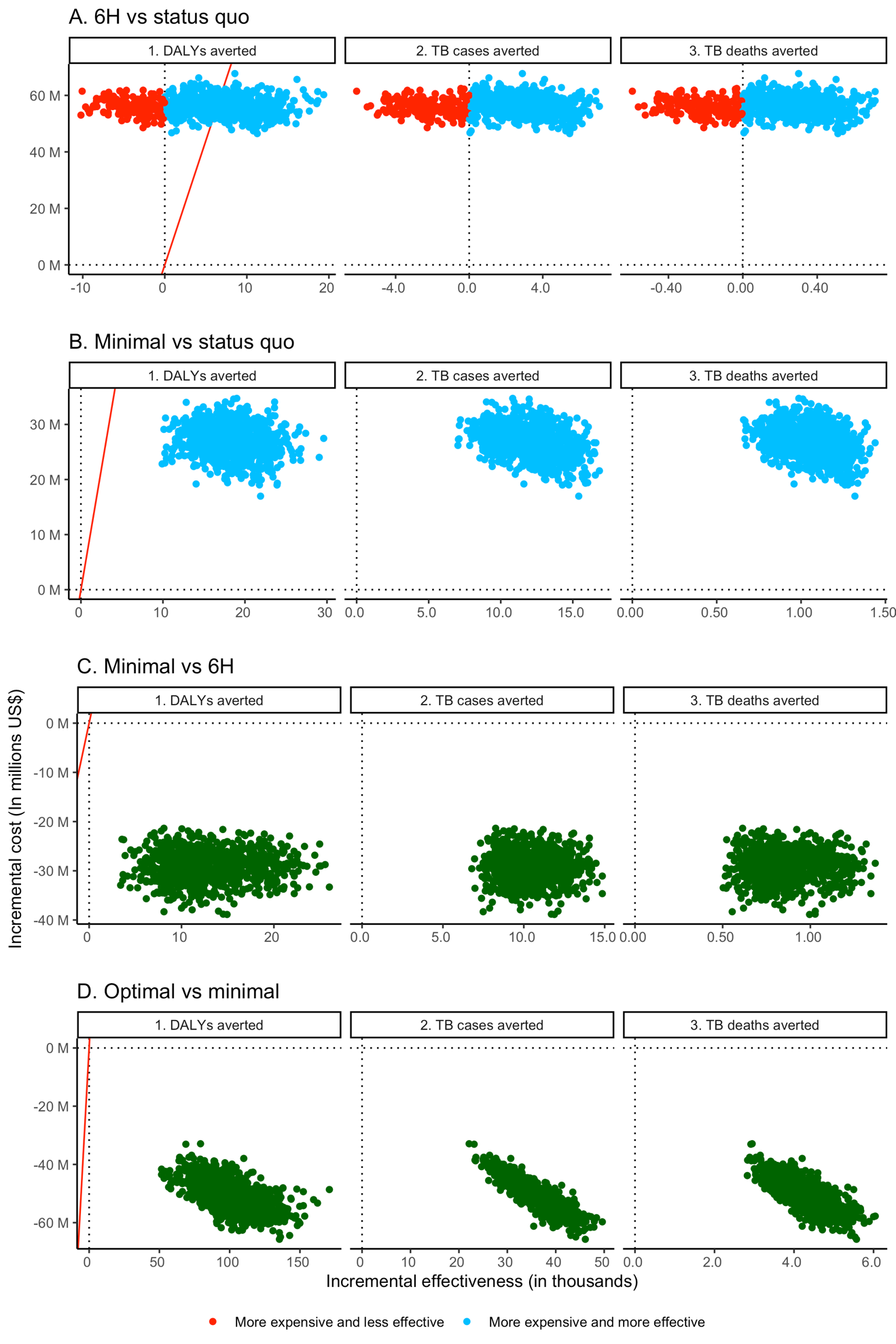


** Results are discounted at a 3% annual rate*

*(A) 6 months daily isoniazid (6H) vs. status quo. (B) Minimal regimen vs. status quo. (C) Minimal regimen vs. 6H. (D) Optimal vs. minimal regimen.*

*On the x-axis, negative values indicate poorer health outcomes. On the y-axis, negative values indicate cost savings. The red lines correspond to the willingness-to-pay threshold per DALY averted ($8 786/DALY averted). 6H = six months of daily isoniazid.*

**References**

1. Steffen RE, Caetano R, Pinto M, Chaves D, Ferrari R, Bastos M, et al. Cost-effectiveness of Quantiferon®-TB Gold-in-Tube versus tuberculin skin testing for contact screening and treatment of latent tuberculosis infection in Brazil. PloS one. 2013;8(4):e59546. <https://www.ncbi.nlm.nih.gov/pmc/articles/PMC3617186/pdf/pone.0059546.pdfhttps://www.ncbi.nlm.nih.gov/pmc/articles/PMC3617186/pdf/pone.0059546.pdf>.

2. Bozzani FM, Mudzengi D, Sumner T, Gomez GB, Hippner P, Cardenas V, et al. Empirical estimation of resource constraints for use in model-based economic evaluation: an example of TB services in South Africa. Cost Eff Resour Alloc. 2018;16:27. <https://doi.org/10.1186/s12962-018-0113-z>.

3. Stop TB Partnership. Global drug facility. 2016. <http://www.stoptb.org/gdf/drugsupply/pc2.asp?CLevel=2&CParent=4http://www.stoptb.org/gdf/drugsupply/pc2.asp?CLevel=2&CParent=4>.

4. Pinto M, Steffen R, Cobelens F, van den Hof S, Entringer A, Trajman A. Cost-effectiveness of the Xpert® MTB/RIF assay for tuberculosis diagnosis in Brazil. The International Journal of Tuberculosis and Lung Disease. 2016;20(5):611-8. <https://doi.org/10.5588/ijtld.15.0455>.

5. Steffen R, Menzies D, Oxlade O, Pinto M, de Castro AZ, Monteiro P, et al. Patients' costs and cost-effectiveness of tuberculosis treatment in DOTS and non-DOTS facilities in Rio de Janeiro, Brazil. PLoS One. 2010;5(11):e14014. <https://www.ncbi.nlm.nih.gov/pmc/articles/PMC2984447/pdf/pone.0014014.pdfhttps://www.ncbi.nlm.nih.gov/pmc/articles/PMC2984447/pdf/pone.0014014.pdf>.

6. Sumner T, Bozzani F, Mudzengi D, Hippner P, Houben RM, Cardenas V, et al. Estimating the Impact of Tuberculosis Case Detection in Constrained Health Systems: An Example of Case-Finding in South Africa. Am J Epidemiol. 2019;188(6):1155-64. <https://doi.org/10.1093/aje/kwz038>.

7. Vassall A, Siapka M, Foster N, Cunnama L, Ramma L, Fielding K, et al. Cost-effectiveness of Xpert MTB/RIF for tuberculosis diagnosis in South Africa: a real-world cost analysis and economic evaluation. Lancet Glob Health. 2017;5(7):e710-e9. <https://doi.org/10.1016/S2214-109X(17)30205-X>.

8. Pooran A, Theron G, Zijenah L, Chanda D, Clowes P, Mwenge L, et al. Point of care Xpert MTB/RIF versus smear microscopy for tuberculosis diagnosis in southern African primary care clinics: a multicentre economic evaluation. Lancet Glob Health. 2019;7(6):e798-e807. <https://doi.org/10.1016/S2214-109X(19)30164-0>.

9. Durovni B, Saraceni V, van den Hof S, Trajman A, Cordeiro-Santos M, Cavalcante S, et al. Impact of Replacing Smear Microscopy with Xpert MTB/RIF for Diagnosing Tuberculosis in Brazil: A Stepped-Wedge Cluster-Randomized Trial. PLOS Medicine. 2014;11(12):e1001766. 10.1371/journal.pmed.1001766. <https://doi.org/10.1371/journal.pmed.1001766>.

10. Theron G, Zijenah L, Chanda D, Clowes P, Rachow A, Lesosky M, et al. Feasibility, accuracy, and clinical effect of point-of-care Xpert MTB/RIF testing for tuberculosis in primary-care settings in Africa: a multicentre, randomised, controlled trial. Lancet. 2014;383(9915):424-35. <https://doi.org/10.1016/S0140-6736(13)62073-5>.

11. DATASUS. hospitalization costs. 2016. <http://www2.datasus.gov.brhttp://www2.datasus.gov.br>.

12. Pooran A, Pieterson E, Davids M, Theron G, Dheda K. What is the Cost of Diagnosis and Management of Drug Resistant Tuberculosis in South Africa? PLOS ONE. 2013;8(1):e54587. 10.1371/journal.pone.0054587. <https://doi.org/10.1371/journal.pone.0054587>.

13. Programa_Nacional_de_Controle_da_Tuberculose. Manual de Recomendações para o Controle da Tuberculose no Brasil. Ministério da Saúde. 2011;2010.

14. Trajman A, Bastos ML, Belo M, Calaça J, Gaspar J, Dos Santos AM, et al. Shortened first-line TB treatment in Brazil: potential cost savings for patients and health services. BMC Health Serv Res. 2016;16:27. <https://doi.org/10.1186/s12913-016-1269-x>.

15. Department of Health RoSA. National Tuberculosis Management Guidelines 20142014. <http://www.health.gov.za/index.php/shortcodes/2015-03-29-10-42-47/2015-04-30-08-18-10/2015-04-30-08-23-21>. 30 Oct 2020

16. Consortium TDMA. Market assessment of tuberculosis diagnostics in South Africa, 2012-2013. The international journal of tuberculosis and lung disease: the official journal of the International Union against Tuberculosis and Lung Disease. 2015;19(2):216.
