# Supplementary material for: Scaling up target regimens for tuberculosis preventive treatment in Brazil and South Africa: An analysis of costs and cost-effectiveness
Source: PLoS Med. 2022 Jun 13;19(6):e1004032. doi: 10.1371/journal.pmed.1004032 (PMC9239450; doi:10.1371/journal.pmed.1004032)
Supplement: S1 Modeling Methods — (DOCX) [file pmed.1004032.s005.docx]

**Scaling-up Target Regimens for Tuberculosis Preventive Treatment in Brazil and South Africa: An Analysis of Costs and Cost-Effectiveness**

**S1. Modeling methods**

TB transmission model

The epidemiological projections underlying our economic evaluation were produced using a dynamic TB transmission model. We used a compartmental, deterministic model to represent the acquisition of TB, progression to disease, recruitment into preventive therapy and into further TB care for active disease. A schematic representation of the model can be seen in Fig.

Fig: Schematic flowchart of the TB transmission model


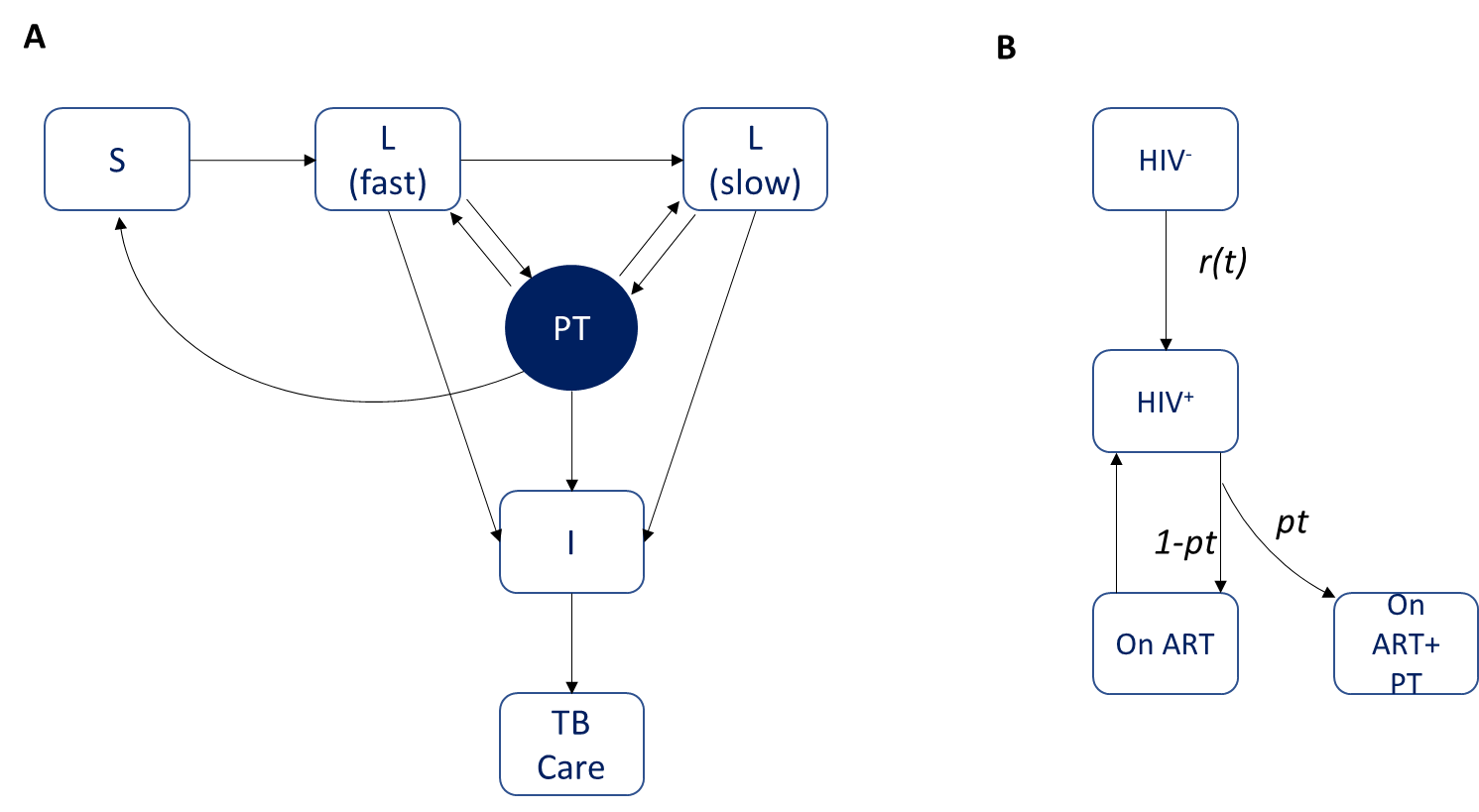


**Fig.** **Schematic of TB compartmental model:** **A**) Acquisition of TB for susceptible individuals (S) is followed by a stage of latent TB infection of fast progression (L fast). Most transitions into active TB (I) from this stage occur in the first two years, otherwise individuals move into a latent stage of slow progression (L slow) where transition into active disease is very slow or even unlikely during a lifetime. From both latent compartments, individuals can be recruited into preventive therapy (PT). This recruitment rate is modelled to reflect full uptake of WHO prevention guidelines. During PT, transition into active TB is dimmed by the effect of the preventive regimen. After PT administration (regimen duration), individuals return to the natural track of TB progression, but some benefit from the fully curative effect of PT, hence the arrow from PT to S. Finally, individuals with active TB (I) can be diagnosed and treated according to the local capacity and coverage of the specific countries modelled. In panel **B)** a schematic of transitions in the HIV status dimension of the model. HIV negative individuals acquire HIV at a time-varying rate r(t) (time trend taken from UNAIDS country fact sheets). HIV+ individuals are detected and recruited into ART at a rate that satisfies local trends (during calibration), and a fraction *pt* will be set on TB preventive therapy at the time of starting ART. This HIV dimension of the model applies to every other compartment of the TB transitions explained in panel A.

We model transmission of drug susceptible and drug resistant strains, and the effect of preventive and treatment regimens effectiveness and durations accordingly. Also, a structure by HIV status is part of the model dimensions. The model is not stratified by age, and the demographic components of the model reflect the average mortality, and life expectancy in the overall population.

Model Parameters

| Parameter | Symbol | Value | | | Source/Notes |
| --- | --- | --- | --- | --- | --- |
|  | | **South Africa** | | **Brazil** |  |
| TB natural history | | | | | |
| Mean rate of transmission per DS-TB case | $\beta_{ds}$ | 5.1 (95% CrI 2.3 – 10.6) | | 37 (95% CrI 22 – 49) | Model estimate |
| Mean rate of transmission per DR-TB case | $\beta_{dr}$ | 4.3 (95% CrI 2 – 7.7) | | 16 (95% CrI 10 – 25) | Model estimate |
| TB infectiousness in $\boldsymbol{HIV}^{\boldsymbol{+}}$ relative to $\boldsymbol{HIV}^{\boldsymbol{-}}$ | $\alpha$ | 0.77(95% CrI 0.61 – 0.99) | | 0.79 (95% CrI 0.6 – 0.98) | Model estimate |
| Breakdown to active disease in slow progressors | $\gamma^{slow}$ | 0.000594 | | | Menzies et al.(1) |
| Breakdown to active disease in fast progressors | $\gamma^{fast}$ | 0.19 (95% CrI 0.07 – 0.6) | | 0.04 (95% CrI 0.02 – 0.07) | Model estimate |
| Rate of transition to the slow latent compartment | $\delta$ | 0.87 | | | Menzies et al. (1) |
| Increased progression to TB in $\boldsymbol{HIV}^{\boldsymbol{+}}$ relative to $\boldsymbol{HIV}^{\boldsymbol{-}}$ | $\varepsilon$ | 26 | | | Getahun et al. (2) |
| Relapse, per-capita hazard rates | $\zeta_{g}$ | *g* = 0; relapse following treatment completion | | 0.032 | Driver et al.(3), Thomas et al(4). Menzies et al.(5) |
|  |  | *g* = 1; relapse following treatment default | | 0.14 |  |
|  |  | *g* = 2; relapse >2 years after treatment | | 0.0015 |  |
| ‘Stabilisation’ of relapse risk following treatment | $\eta$ | 0.5 | | | Based on Thomas et al: most relapse occurs in first 2 yr after treatment. |
| TB mortality rate | $\mu^{(tb)}$ | 0.16 (95% CrI 0.12 – 0.2) | | 0.08 (95% CrI 0.08 – 0.11) | Specified together to yield ~50% cure, ~50% mortality in average of 3 years. Tiemersma et al. (6) |
| Spontaneous cure | $\theta$ | 0.15 [0.14 –0.18] | | |  |
| Relative Risk of TB mortality in HIV^+^ | RR^(h+)^ | 3.3 (95% CrI 1.25-5.8) | | 3.3 (95% CrI 1.7-5.6) | Model estimate |
| Reduced susceptibility from past infection | $\iota$ | [0.25 –0.75] | | | Assumed range (uniform distribution) |
| Health system | | | | | |
| Per-capita rate of initial presentation to care | $\kappa$ | 2.8 (95% CrI 1 – 5.9) | | 10 (95% CrI 7 – 12) | Model estimate: corresponds to mean initial patient delay of 4.6 months (95% CrI 3.6 – 6) |
| Rate of Interval between care-seeking episodes (factor increase relative to initial careseeking) | $\nu$ | 2 | | | Model assumption corresponds to delay decrease of 50% between careseeking episodes |
| Treatment initiation delay | $\xi$ | 52 | | | Sreeramareddy et al.(7); corresponds a mean treatment delay of 1 week |
| Probability of diagnosis per patient-provider interaction | $o_{h}$ | h=0; 0.7 (95% CrI 0.51 – 0.94) | | h=0; 0.85 (95% CrI 0.52 – 0.97) | Model estimate |
|  |  | h=1; 0.73 (95% CrI 0.52 – 0.97) | | h=1; 0.73 (95% CrI 0.51 – 0.96) |  |
| Probability of rapid DST during diagnostic attempt | $\varpi$ | 71% | | 33% | (8) |
| Rapid DST sensitivity | $\rho^{(xp)}$ | 90% | | | (9) |
| Smear test sensitivity | $\rho^{(sm)}$ | 80% | | | (9) |
| Treatment initiation probability | $\varrho$ | 0.72 (95% CrI 0.51 – 0.96) | | 0.83 (95% CrI 0.54 – 0.97) | Model estimate |
| Overall diagnostic probability | $\epsilon_{s}$ | $\epsilon_{s}=o_{h}\left( \varpi\rho^{(xp)}+\left( 1-\varpi\right)\rho^{(sm)} \right), for s=0$ | | | Reflects the probability of diagnosis given is a DS strain |
|  |  | $\epsilon_{s}=o_{h}\varpi\rho^{(xp)}, for s=1$ | | | Reflects the probability of diagnosis given is a DR strain |
| Proportion completing first line treatment | $\sigma^{(fl)}$ | 84.19% | | 71.02% | (10) |
| Proportion completing second line treatment | $\sigma^{(sl)}$ | 55.2% | | 61.2% | (10) |
| Proportion failing first line treatment | $x^{(fl)}$ | 0.38% | | 0.05% | (10) |
| Proportion failing second line treatment | $x^{(sl)}$ | 3.27% | | 5.31% | (10) |
| Proportion lost to follow-up in first line treatment | $y^{(fl)}$ | 8.3% | | 10.7% | (10) |
| Proportion lost to follow-up in second line treatment | $y^{(sl)}$ | 20.1% | | 20.9% | (10) |
| Proportion dying in first line treatment | $j^{(fl)}$ | 7.1% | | 7.8% | (10) |
| Proportion dying in second line treatment | $j^{(sl)}$ | 21.4% | | 11.2% | (10) |
| First line treatment duration | $\tau^{(fl)}$ | 2 y^-1^ | | | Corresponds to 6 month duration for standard first-line regimen. WHO (11) |
| Second line treatment duration | $\tau^{(sl)}$ | 0.5 y^-1^ | | | Corresponds to 24 month duration for standard second-line regimen. WHO (11) |
| Probability of cure after first line completion | $\varsigma^{(fl)}$ | $\varsigma^{(fl)}=\frac{\sigma^{(fl)}}{\left( \sigma^{(fl)}+x^{(fl)} \right)}$ | | | |
| Probability of cure after second line completion | $\varsigma^{(sl)}$ | $\varsigma^{(sl)}=\frac{\sigma^{(sl)}}{\left( \sigma^{(sl)}+x^{(sl)} \right)}$ | | | |
| First line default hazard | $\varphi^{(fl)}$ | $\varphi^{(fl)}=y^{(fl)}\frac{\tau^{(fl)}}{\left( \sigma^{(fl)}+x^{(fl)} \right)}$ | | | |
| Second line default hazard | $\varphi^{(sl)}$ | $\varphi^{(sl)}=y^{(sl)}\frac{\tau^{(sl)}}{\left( \sigma^{(sl)}+x^{(sl)} \right)}$ | | | |
| Mortality hazard during first line treatment | $\mu^{(fl)}$ | $\mu^{(sl)}=j^{(fl)}\frac{\tau^{(fl)}}{\left( \sigma^{(fl)}+x^{(fl)} \right)}$ | | | |
| Mortality hazard during first line treatment | $\mu^{(sl)}$ | $\mu^{(sl)}=j^{(sl)}\frac{\tau^{(sl)}}{\left( \sigma^{(sl)}+x^{(sl)} \right)}$ | | | |
| Rate of recruitment into ART | $\Pi$ | 4.4 (95% CrI 3.3 – 6.8) | 6.2 (95% CrI 3.8 – 9.3) | | Model estimate |
| Fraction of new ART starters enrolled in PT | A | 0.7 (95% CrI 0.54 – 0.82) | | | Model estimate |
| Preventive therapy (parameters for a baseline 6 months course of isoniazid -6H) | | | | | |
| PT regimen duration (months) | $\Gamma$ | 6 | | | (12) |
| Rate of transition over half-course of PT | $m$ | $m= \frac{\Gamma}{2}$ | | | Assumption |
| Ease of adherence (completion) | $\Theta$ | 70% | | | (13) |
| PT default hazard | $d$ | $d=\frac{12\left( 1-\Theta\right)}{\Gamma\Theta}$ | | |  |
| Forgiveness of non-completion | $f$ | 25% | | | Assumption |
| Waning of PT effect | $g$ | $g=\frac{12}{60}$ | | | To reflect an annualised rate of 60 months average protection |
| Suppression of reactivation effect | $e$ | 70% | | | (14) |
| Curative effect of PT | c | 0% | | | Assumption |
| PT *Rif* resistance barrier | b | 100% | | | Assumption: 6H regimen cannot induce Rif resistance |
| Demographics | | | | | |
| Birth rate | $B$ | 0.013 | | 0.008 | WHO (GHO) (15)– adjusted to yield annual population growth from 1970 |
| Background mortality rate | $\mu$ | 0.016 | | 0.013 | WHO (GHO)(15), corresponds to mean life expectancy |
| Ratio of HIV-infected to HIV-uninfected case fatality rates for TB | 1.14 | | | | Corbett et al. 2003 (16) |

**Note: Symbols are as used in the mathematical model. Numbers in brackets show 95% uncertainty intervals.**

Model Calibration

Epidemiological parameters were estimated in a Bayesian calibration framework, using MCMC and a Metropolis-Hastings algorithm. Calibration targets for each country come from WHO reports and reflect incidence, mortality, and HIV/TB burden in each setting (Table). The model was calibrated against data for the years 2012 and 2019, to capture the epidemiological trend in most recent years.

Mortality and DALY estimation

DALYs are constructed using simulation output, with the usual YLL + YLD formula. Years of Life Lost (YLL) prematurely due to disease are calculated yearly by multiplying the number of deaths caused by TB, by the life expectancy in each country. As the model does not include an age structure, the life expectancy is taken to be that of an average person in the population. Years of Life lost to Disability (YLD) are calculated yearly as the total number of people living with the disease (TB) multiplied by a disability weight. We used the estimated weights by Solomon et al (Lancet Global Health 2015;3(11):E712-E723), for pulmonary TB cases without HIV (0.33), and with HIV (0.48).

Model projections

Samples from the posterior distribution resulting from the model calibration were used to run instances of the model for projecting incidence, prevalence, and mortality until 2035. These trajectories were allowed to vary according to different interventions (variations in preventive regimens). Uncertainty in our results arise from the epidemiological model calibration and are presented as 95% Credible intervals.

**Table: Target data for model calibration. Numbers in parentheses represent uncertainty intervals.**

| **Country** | **TB Incidence per 100K** | | **MDR-TB incidence per 100K** | **TB Incidence per 100K (HIV+ only)** | **TB Mortality per 100K (HIV-)** | **TB Mortality per 100K (HIV+)** | **TB notification rate per 100K** | **Proportion of** $\boldsymbol{HIV}^{\boldsymbol{+}}$**on ART (%)*** | **Proportion of those on ART that have received TPT (%)** |
| --- | --- | --- | --- | --- | --- | --- | --- | --- | --- |
|  | **2012** | **2019** | **2019** | **2019** | **2019** | **2019** | **2019** | **2019** | **2019** |
| ***South Africa*** | 1160  (809-1580) | 615  (427-835) | 24  (14-34) | 357  (248-486) | 38  (36-40) | 62  (25-115) | 358  (286-429) | 71  (65-75) | 69  (55-82) |
| ***Brazil*** | 44  (38-51) | 46  (39-53) | 1.2  (0.9-1.5) | 5.1  (4.3-6) | 2.3  (2.2-2.4) | 0.87  (0.65-1.1) | 40  (32-48) | 69  (48-90) | 10  (8-12) |

++ *For calibration purposes* *we use uncertainty intervals as estimated by WHO.*

** Source: UNAIDS AIDSinfo Online Database [http://aidsinfoonline.org/devinfo/libraries/aspx/Home.aspx ]*

*All other data retrieved from WHO TB country profiles [https://worldhealthorg.shinyapps.io/tb_profiles/], except for proportion on ART who received TPT in Brazil which is derived from Durovni et al [doi:* *10.1097/01.aids.0000391022.95412.a6]*

References

1. Menzies NA, Wolf E, Connors D, Bellerose M, Sbarra AN, Cohen T, et al. Progression from latent infection to active disease in dynamic tuberculosis transmission models: a systematic review of the validity of modelling assumptions. Lancet Infect Dis [Internet]. 2018 Aug 1 [cited 2021 Feb 25];18(8):e228–38. Available from: https://pubmed.ncbi.nlm.nih.gov/29653698/

2. Getahun H, Gunneberg C, Granich R, Nunn P. HIV infection-associated tuberculosis: the epidemiology and the response. Clin Infect Dis [Internet]. 2010;50 Suppl 3:S201-7. Available from: https://www.ncbi.nlm.nih.gov/pubmed/20397949

3. Driver CR, Munsiff SS, Li J, Kundamal N, Osahan SS. Relapse in persons treated for drug-susceptible tuberculosis in a population with high coinfection with human immunodeficiency virus in New York City. Clin Infect Dis [Internet]. 2001;33(10):1762–9. Available from: https://www.ncbi.nlm.nih.gov/pubmed/11595988

4. Thomas A, Gopi PG, Santha T, Chandrasekaran V, Subramani R, Selvakumar N, et al. Predictors of relapse among pulmonary tuberculosis patients treated in a DOTS programme in South India. Int J Tuberc Lung Dis [Internet]. 2005;9(5):556–61. Available from: https://www.ncbi.nlm.nih.gov/pubmed/15875929

5. Menzies D, Benedetti A, Paydar A, Martin I, Royce S, Pai M, et al. Effect of duration and intermittency of rifampin on tuberculosis treatment outcomes: a systematic review and meta-analysis. PLoS Med [Internet]. 2009;6(9):e1000146. Available from: https://www.ncbi.nlm.nih.gov/pubmed/19753109

6. Tiemersma EW, van der Werf MJ, Borgdorff MW, Williams BG, Nagelkerke NJ. Natural history of tuberculosis: duration and fatality of untreated pulmonary tuberculosis in HIV negative patients: a systematic review. PLoS One [Internet]. 2011;6(4):e17601. Available from: https://www.ncbi.nlm.nih.gov/pubmed/21483732

7. Sreeramareddy CT, Qin ZZ, Satyanarayana S, Subbaraman R, Pai M. Delays in diagnosis and treatment of pulmonary tuberculosis in India: a systematic review. Int J Tuberc Lung Dis [Internet]. 2014;18(3):255–66. Available from: https://www.ncbi.nlm.nih.gov/pubmed/24670558

8. WHO | Tuberculosis country profiles [Internet]. [cited 2020 Apr 24]. Available from: https://www.who.int/tb/country/data/profiles/en/

9. Steingart KR, Ng V, Henry M, Hopewell PC, Ramsay A, Cunningham J, et al. Sputum processing methods to improve the sensitivity of smear microscopy for tuberculosis: a systematic review [Internet]. Vol. 6, Lancet Infectious Diseases. Elsevier; 2006 [cited 2021 Mar 1]. p. 664–74. Available from: http://www.thelancet.com/article/S1473309906706028/fulltext

10. WHO. Tuberculosis data [Internet]. [cited 2021 Mar 2]. Available from: https://www.who.int/teams/global-tuberculosis-programme/data

11. WHO | Guidelines for treatment of tuberculosis [Internet]. WHO. Geneva: World Health Organization; 2015 [cited 2021 Feb 25]. Available from: https://www.who.int/tb/publications/2010/9789241547833/en/

12. WHO | Latent TB Infection : Updated and consolidated guidelines for programmatic management [Internet]. [cited 2020 Apr 23]. Available from: https://www.who.int/tb/publications/2018/latent-tuberculosis-infection/en/

13. Alsdurf H, Hill PC, Matteelli A, Getahun H, Menzies D. The cascade of care in diagnosis and treatment of latent tuberculosis infection: a systematic review and meta-analysis. Lancet Infect Dis. 2016 Nov 1;16(11):1269–78.

14. Whalen CC, Johnson JL, Okwera A, Hom DL, Huebner R, Mugyenyi P, et al. A trial of three regimens to prevent tuberculosis in ugandan adults infected with the human immunodeficiency virus. N Engl J Med. 1997 Sep 18;337(12):801–8.

15. WHO. Global Health Observatory [Internet]. [cited 2021 Feb 25]. Available from: https://www.who.int/data/gho

16. Corbett EL, Watt CJ, Walker N, Maher D, Williams BG, Raviglione MC, et al. The growing burden of tuberculosis: global trends and interactions with the HIV epidemic. Arch Intern Med [Internet]. 2003;163(9):1009–21. Available from: https://www.ncbi.nlm.nih.gov/pubmed/12742798
